# Fault Point Search with Obstacle Avoidance for Machinery Diagnostic Robots Using Hierarchical Fuzzy Logic Control

**DOI:** 10.3390/s25196127

**Published:** 2025-10-03

**Authors:** Rui Mu, Ryojun Ikeura, Hongtao Xue, Chengxiang Zhao, Peng Chen

**Affiliations:** 1 Graduate School of Engineering, Mie University, Tsu 514-8507, Japan; 424de51@m.mie-u.ac.jp (R.M.); ikeura@mach.mie-u.ac.jp (R.I.); 2 School of Automotive and Traffic Engineering, Jiangsu University, Zhenjiang 212013, China; xueht@ujs.edu.cn; 3 School of Vehicle and Mobility, Tsinghua University, Beijing 100084, China; zhaocx0221@126.com; 4 Graduate School of Bioresources, Mie University, Tsu 514-8507, Japan

**Keywords:** diagnostic robot, fuzzy control, fault point searching, robot navigation, obstacle avoidance, multi-objective planning

## Abstract

Higher requirements have been placed on fault detection for continuously operating machines in modern factories. Manual inspection faces challenges related to timeliness, leading to the emergence of autonomous diagnostic robots. To overcome the safety limitations of existing diagnostic robots in factory environments, a hierarchical fuzzy logic-based navigation and obstacle avoidance algorithm is proposed in this study. The algorithm is constructed based on zero-order Takagi–Sugeno type fuzzy control, comprising subfunctions for navigation, static obstacle avoidance, and dynamic obstacle avoidance. Coordinated navigation and equipment protection are achieved by jointly considering the information of the fault point and surrounding equipment. The concept of a dynamic safety boundary is introduced, wherein the normalized breached level is used to replace the traditional distance-based input. In the inference process for dynamic obstacle avoidance, the relative speed direction is additionally considered. A Mamdani-type fuzzy inference system is employed to infer the necessity of obstacle avoidance and determine the priority target for avoidance, thereby enabling multi-objective planning. Simulation results demonstrate that the proposed algorithm can guide the diagnostic robot to within 30 cm of the fault point while ensuring collision avoidance with both equipment and obstacles, enhancing the completeness and safety of the fault point searching process.

## 1. Introduction

Industrial automation is a core driver of modern manufacturing, improving efficiency and quality while accelerating due to rising labor costs and an aging workforce. However, failure detection, diagnosis, and maintenance still require substantial human effort, and continuous operation often prevents timely manual inspection. This creates a cost–risk imbalance between maintaining standby personnel and tolerating delayed fault responses. To mitigate this gap, autonomous diagnostic robots have been deployed as always-on safeguards that patrol facilities and respond to faults continuously.

The functionality of a diagnostic robot can be divided into four main components [1]: path tracking for factory patrol, local path planning for fault machine searching, manipulator control for precise fault point localization, and advanced fault diagnosis.

Among these, local path planning for fault point searching is pivotal, since robots must navigate in partially known, structurally complex spaces that may contain static and dynamic obstacles. Effective realization therefore requires a tightly integrated and dynamically balanced scheme that coordinates navigation and obstacle avoidance under uncertainty.

### 1.1. Related Works

At present, various fault point recognition algorithms have been extensively studied and applied, including vision-based methods [2,3], infrared sensor-based methods [4], and acoustic signal-based methods [5,6,7]. Meanwhile, a range of navigation algorithms has also been proposed [8,9,10]. For diagnostic robots, the key lies in effectively integrating target recognition and localization results into the navigation system, thereby enabling coordinated control of perception and motion. Zhang et al. [11] constructed a map using 2D LiDAR and performed local localization through Adaptive Monte Carlo Localization. A lightweight YOLOv5 deep neural network model was employed for button target detection, and the ROS navigation stack was used to implement local path planning. Yao et al. [12] employed image recognition techniques to identify equipment anomalies and performed local path planning based on a pre-constructed global map and environmental information obtained from ultrasonic sensors. However, map-dependent navigation introduces additional workload when environmental updates are required. Wu et al. [5] utilized cameras and ultrasonic sensors for target recognition and employed PID control for robot navigation. Wang et al. [13] conducted fault diagnosis within a cloud computing environment and optimized the robot’s path using an improved ant colony optimization algorithm. Although the aforementioned studies have made notable progress in navigation functionality, safety considerations in practical factory environments remain insufficient. In particular, equipment protection and the avoidance of both static and dynamic obstacles are critical safety concerns that must be addressed during fault point search and navigation.

Obstacle avoidance algorithms for robots have been extensively studied by numerous researchers, leading to the development of a variety of approaches. Rule-based methods, such as support vector machines [14] and PID-based strategies [15], offer relatively straightforward solutions but exhibit limitations when dealing with the uncertainty and ambiguity commonly present in complex factory environments. Optimization-based methods, including those based on improved particle swarm optimization [16], nonlinear model predictive control [17], and enhanced artificial potential fields [18], typically require accurate model design and may incur substantial computational costs. Artificial intelligence-based methods, such as deep learning approaches [19], the dynamic window approach optimized via deep deterministic policy gradients [20], and self-learning methods based on fuzzy neural networks [21], generally rely on large volumes of high-quality training data to ensure model performance. Although the algorithms have played a significant role in advancing robotic obstacle avoidance research, factory environments are often characterized by unstructured layouts and dynamic changes. For instance, stacked materials and other types of robots (e.g., those used for transporting materials or components) may act as temporary static or dynamic obstacles. Additionally, low-illumination conditions are common due to automated operations. These factors not only increase the difficulty and cost of detailed environmental modeling and data acquisition but also substantially hinder the ability of perception systems to obtain accurate information. As a result, the effectiveness of control methods that rely on precise environmental models is greatly diminished in practical applications.

Compared to other control methods, fuzzy logic control (FLC) offers significant advantages in handling uncertainty, mimicking human reasoning, and enabling real-time control in resource-constrained environments [22,23]. It also demonstrates greater robustness to sensor noise and environmental fluctuations. In practice, diagnostic robots typically do not require high-precision trajectory tracking and are afforded greater motion tolerance and flexibility. Within the context of ‘natural driving within a permitted trajectory range,’ fuzzy control is particularly well-suited for implementing human-like autonomous navigation strategies due to its flexibility, simplicity, and reliability. Consequently, it has been widely applied in diagnostic robot navigation with multi-objective motion control. Mohanty et al. [24] employs a conventional Takagi–Sugeno (T–S) fuzzy controller with a grid-partitioned rule base and fuses static obstacle avoidance, wall following, and navigation to achieve real-time navigation and collision avoidance in unknown environments. Wondosen et al. [25] proposed a single fuzzy logic navigation controller for outdoor unknown environments with static and dynamic obstacles. Similarly, integrating behavioral strategies directly into the rule set can easily lead to the curse of dimensionality. Kumar et al. [26] proposed a Takagi–Sugeno–Kang fuzzy control scheme that combines a navigation controller and an obstacle avoidance controller via weighted fusion to generate linear and angular velocity commands. Faisal et al. [27] proposed a dual fuzzy control scheme that combines navigation and obstacle avoidance switches based on frontal obstacle detection. Hachani et al. [28] proposed an interval T–S fuzzy control scheme with navigation and obstacle avoidance, using Karnik–Mendel type reduction and a simple obstacle detection switch to select wheel speed outputs. Pandey et al. [29] proposed a fuzzy multilayer decision framework that switches between navigation and obstacle avoidance upon obstacle detection. In multi-obstacle scenes, it prioritizes avoidance by focusing on the nearest obstacles and on dynamic trajectories, and it uses a fuzzy logic controller to compute a risk score for strategy selection. Most multi-objective approaches adopt identical strategies for both static and dynamic obstacle avoidance, without fully accounting for their shared and distinct spatiotemporal characteristics and motion patterns, thereby limiting adaptability to dynamic environments. Moreover, the switching between navigation and obstacle avoidance functions is typically triggered by obstacle detection, which limits the ability to achieve dynamic coordination between task objectives and environmental risks. In addition, in complex scenarios where multiple obstacles simultaneously pose potential threats, an effective mechanism for evaluating obstacle prioritization is still lacking, which compromises the specificity and timeliness of avoidance decisions.

### 1.2. Contributions

To address the limitations identified in existing diagnostic robot research and achieve a balance between fault point searching and both static and dynamic obstacle avoidance, this study proposes a hierarchical T–S FLC [30]-based algorithm for multi-objective fault point search, navigation, and obstacle avoidance. The algorithm adopts a modular path planning architecture comprising a navigation subsystem, a static obstacle avoidance subsystem, and a dynamic obstacle avoidance subsystem. The coordination and switching mechanisms among these functional modules are also examined.

Abnormal fault sounds are acquired using a microphone array, and the fault point azimuth is inferred through T–S fuzzy inference. Combined with distance and orientation information of the surrounding equipment obtained from vision sensors, a zero-order T–S fuzzy inference-based navigation controller is constructed. A safety boundary is constructed, with a normalized breached level introduced as a key input variable to replace traditional obstacle distance. A zero-order T–S FLC-based static obstacle avoidance submodule is designed by integrating obstacle orientation and equipment protection principles. For dynamic obstacles, the safety boundary radius is adaptively enlarged based on the relative speed, and the magnitude of the relative speed is incorporated. A dynamic obstacle avoidance submodule based on zero-order T–S FLC is developed by jointly considering breached level, obstacle orientation, and relative speed direction, alongside equipment protection principles. The necessity for obstacle avoidance is inferred using a Mamdani-type Fuzzy Inference System (FIS) based on the relative spatial relationship between obstacle orientation and fault point direction, along with the obstacle’s motion trend and breached level. The urgency of avoidance for each obstacle is inferred through Mamdani-type fuzzy inference using breached level and motion trend, providing a basis for prioritizing avoidance targets. The main contributions of this paper are summarized below.

1. The distance and orientation of the nearest boundary device are considered by the system, enabling coordinated navigation and equipment protection. Equipment protection is incorporated into the obstacle avoidance modules, further enhancing the safety of the system.
2. A dynamic safety boundary concept is proposed to address the shared distance-based requirements of static and dynamic obstacle avoidance. A normalized breached level is introduced to replace traditional distance inputs, thereby unifying the control strategies for both static and dynamic obstacle avoidance.
3. A Mamdani-type fuzzy inference system is developed to evaluate obstacle avoidance necessity and urgency. This enables dynamic switching between navigation and avoidance functions while also identifying the obstacle that requires the highest avoidance priority.

### 1.3. Paper Organization

The remainder of this paper is organized as follows: the fault point search scenario for diagnostic robots and the corresponding navigation and obstacle avoidance control processes are described in Section 2. The fault point search navigation motion planning method based on T–S FLC is proposed in Section 3. The static and dynamic obstacle avoidance method based on T–S FLC is proposed in Section 4. The inference mechanisms for obstacle avoidance necessity and urgency are detailed in Section 5. The proposed algorithm is validated in Section 6. Finally, this study is concluded in Section 7.

## 2. Fault Point Search Scenario and Control Process

### 2.1. Fault Point Search Scenario

**Figure 1:**
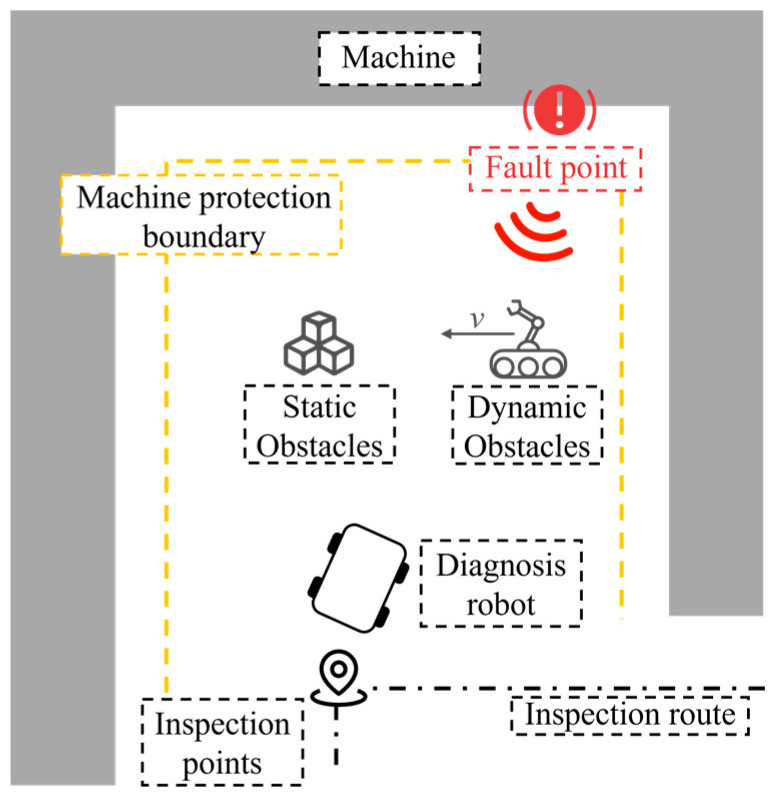
Fault point search scenario.

Figure 1 illustrates the application scenario in which a diagnostic robot performs fault point search in an industrial environment. Equipment failures in factories are often accompanied by abnormal vibrations, which generate perceivable acoustic signals. The diagnostic robot patrols the facility along a predefined inspection route and determines the presence of fault-related acoustic signals at each inspection point. When an abnormal signal is detected, the robot initiates a fault point search toward the direction of maximum sound intensity. During this process, both static and dynamic obstacles may interfere with the robot’s movement. To ensure equipment safety, a protection boundary is incorporated into the control system to constrain the minimum allowable distance between the robot and surrounding equipment. Upon approaching the fault point area, the robot employs a manipulator to perform precise localization and confirmation of the fault.

This study focuses on path planning and multi-objective coordinated control during the movement from the inspection point to the fault point, aiming to ensure efficient fault search while maintaining obstacle avoidance and equipment protection.

### 2.2. Hierarchical T–S FLC-Based Control Framework

**Figure 2:**
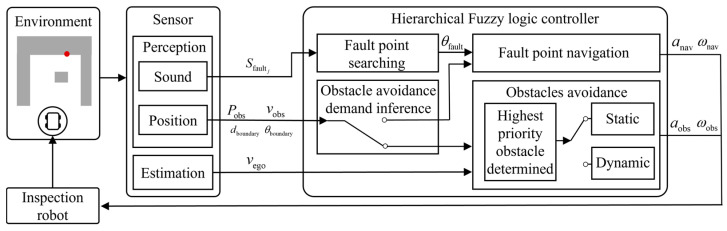
Hierarchical T–S FLC-based control framework.

Figure 2 illustrates the proposed hierarchical T–S fuzzy logic-based control framework. The environment is the same as the scenario mentioned earlier. The robot acquires environmental information through a microphone array and vision sensors. This information includes fault sound intensity $S_{{\mathrm{fault}}_{j}}$, obstacle position *P*_obs_, and speed *v*_obs_, as well as the distance *d*_boundary_ and orientation *θ*_boundary_ of nearby equipment. In addition, the speed of robot *v*_ego_ is obtained through onboard sensors.

Based on the environmental perception results, the system infers the current necessity for obstacle avoidance, enabling dynamic switching between navigation and avoidance modes. In the navigation mode, the fault point azimuth *θ*_fault_ is inferred from multi-directional sound intensity $S_{{\mathrm{fault}}_{j}}$ acquired by the microphone array. This azimuth serves as a key input to the navigation subsystem, which outputs traction acceleration *a*_nav_ and front-wheel steering angle $\omega_{\mathrm{nav}}$ to enable effective tracking of the fault point [1].

When the system determines that obstacle avoidance is required, it switches to avoidance mode. The priority obstacle is identified based on obstacle information, and its dynamic properties are assessed to select the appropriate FLC for avoidance. The selected fuzzy controller then outputs traction acceleration *a*_obs_ and front-wheel steering angle $\omega_{\mathrm{obs}}$, which are applied to the diagnostic robot. The robot continues to interact with the environment, and the control process operates in a closed-loop cycle of perception, inference, decision-making, and execution.

### 2.3. Control Flow of Navigation and Obstacle Avoidance in Fault Point Search

**Figure 3:**
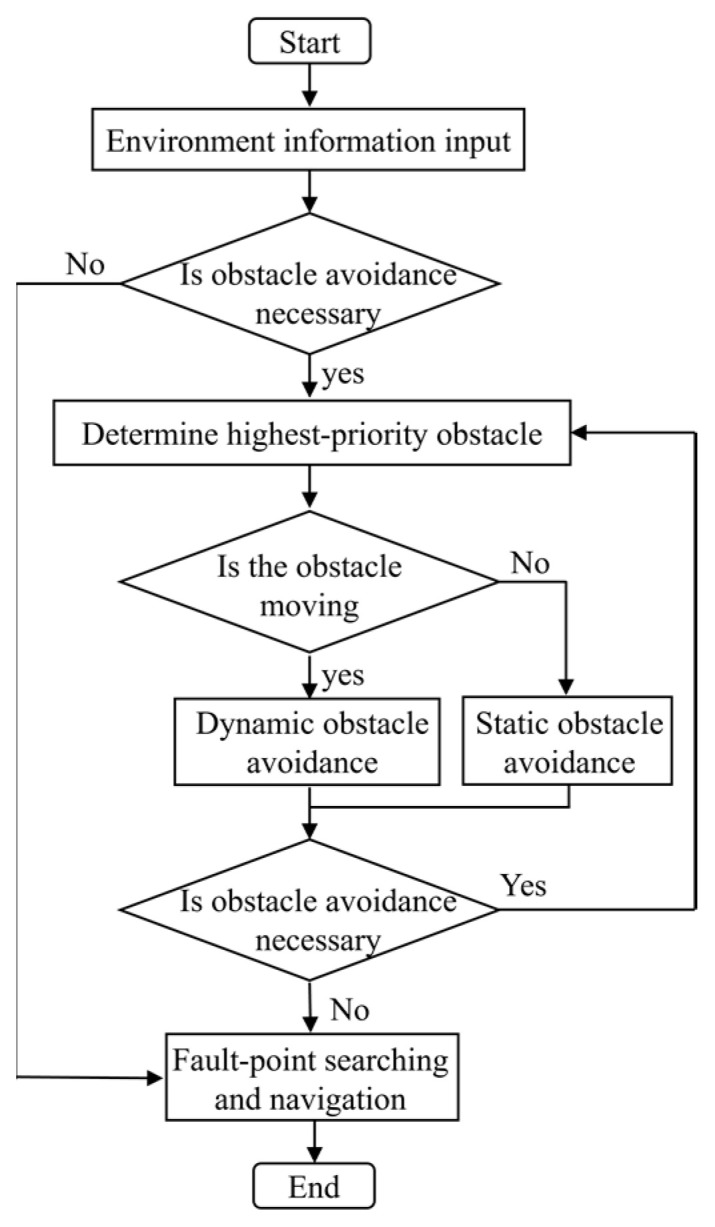
Control flow of navigation and obstacle avoidance in fault point search.

Figure 3 illustrates the control flow of navigation and obstacle avoidance during the fault point search process of the diagnostic robot. The system first acquires the current environmental information and determines whether obstacle avoidance is required. If no avoidance is necessary, the robot remains in navigation mode. If avoidance is deemed necessary, the system identifies the obstacle with the highest priority for avoidance. It then evaluates whether the identified obstacle exhibits dynamic characteristics and based on this assessment, activates the corresponding avoidance submodule. After executing the avoidance maneuver, the system re-evaluates the current necessity for avoidance. If the necessity persists, the avoidance loop is repeated to ensure safe traversal. Once the avoidance condition is resolved, the control flow automatically switches back to the navigation function, allowing the robot to continue progressing toward the fault point.

## 3. Fault Point Search Navigation Using Zero-Order T–S FLC

**Figure 4:**
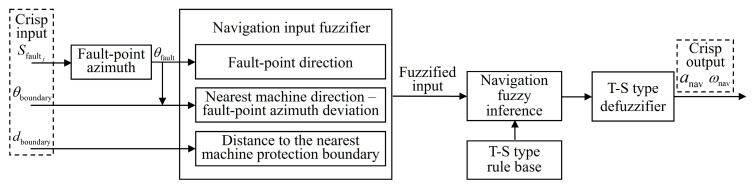
FLC process for navigation.

When no obstacles need to be avoided, fault point search navigation is executed by the robot. The navigation control process is illustrated in Figure 4. The system performs preprocessing and feature extraction on the input scene information: the fault point azimuth *θ*_fault_ is inferred based on the fault sound intensity $S_{{\mathrm{fault}}_{j}}$ collected by the microphone array. The azimuth difference between the nearest equipment and fault point, Δ*θ*_fault-boundary_, is calculated based on *θ*_fault_ and the azimuth of the nearest boundary equipment, *θ*_boundary_. These inputs, along with the distance to the nearest equipment protection boundary *d*_boundary_, are fuzzified and processed through a fuzzy rule base. The outputs are then defuzzified to generate precise control commands for traction acceleration *a*_nav_ and front-wheel steering angle $\omega_{\mathrm{nav}}$.

### 3.1. Kinematic Model of the Diagnostic Robot

The standard bicycle model is adopted as the kinematic model for motion control. The robot’s state vector and input vector are defined as follows:

(1) $$x={\left[X\;Y\;\theta \;v\;\delta \right]}^{T}$$

(2) $$u_{c}={\left[a\;\omega \right]}^{T}$$

where *X* and *Y* denote the position of the robot’s center of mass in the global coordinate frame; *θ* is the heading angle; *v* is the linear speed; δ is the front-wheel steering angle; *a* is the traction acceleration input; and ω is the steering angular speed input. The continuous-time kinematic equations are given as follows:

(3) $$\dot{X}=v\cos\theta$$

(4) $$\dot{Y}=v\sin\theta$$

(5) $$\dot{\theta }=\frac{\nu }{l}\tan\delta$$

(6) $$\dot{v}=a$$

(7) $$\dot{\delta }=\omega$$

where *l* denotes the distance between the front and rear wheel centers.

### 3.2. Fault Point Azimuth Estimation Using Zero-Order T–S Fuzzy Logic

In this study, the diagnostic robot relies on abnormal sounds emitted by faulty equipment as the basis for fault point search, using a microphone array to perceive surrounding acoustic information. However, extracting meaningful features and accurately identifying abnormal fault sounds, along with their spatial intensity distribution in complex acoustic environments, remains a highly challenging research problem, to which many scholars have devoted considerable effort [31]. As this work focuses on motion planning and control of the mobile platform, the modeling process assumes that the sound intensity of the fault source is available, while echoes, noise, and other interference signals are addressed in the signal processing stage.

**Figure 5:**
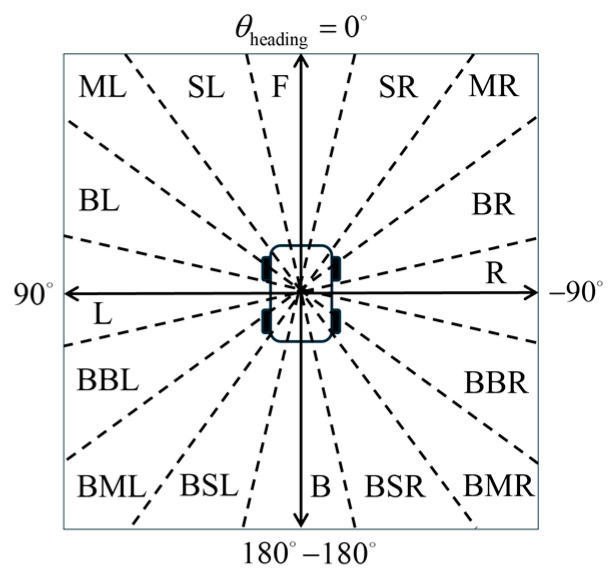
Semantic regions of fault point azimuth.

A microphone array system consisting of 16 microphones is deployed circumferentially around the diagnostic robot to achieve full 360° omnidirectional perception. The directional field of each microphone unit is divided at 22.5° intervals and assigned spatially meaningful directional labels, including the following: Front (F), Slight-Left (SL), Middle-Left (ML), Big-Left (BL), Left (L), Back-Big-Left (BBL), Back-Middle-Left (BML), Back-Slight-Left (BSL), Back (B), Back-Slight-Right (BSR), Back-Middle-Right (BMR), Back-Big-Right (BBR), Right (R), Big-Right (BR), Middle-Right (MR), and Slight-Right (SR), as illustrated in Figure 5. The arrows point to the four directions in ego coordinate system, and the dotted lines represent the semantic divisions for each direction.

Building upon previous studies that employed Mamdani-type fuzzy inference for fault direction estimation [1], T–S fuzzy logic is used for inference. Normalized sound intensity inputs $I_{{\mathrm{fault}}_{j}}$ are constructed based on the abnormal fault sound intensity detected in each direction by the microphone array. The input is defined as follows:

(8) $$I_{{\mathrm{fault}}_{j}}=\frac{S_{{\mathrm{fault}}_{j}}}{S_{{\mathrm{fault}}_{\max}}}$$

where $S_{{\mathrm{fault}}_{j}}$ denotes the fault sound intensity in the *j*th direction, and $S_{{\mathrm{fault}}_{\max}}$ denotes the maximum sound intensity received at the current position of the diagnostic robot. A single-set membership function is designed for the sound intensity of each directional acoustic sensor based on a quadratic function:

(9) $$\omega_{j}=\mu_{I}(I_{{\mathrm{fault}}_{j}})={\left(I_{{\mathrm{fault}}_{j}}\right)}^{2}$$

where $\omega_{j}$ denotes the activation degree of the *j*th rule. A constant output is assigned to each T–S rule as follows:

(10) $$y_{j}=c_{j},c_{j}=\theta_{j}$$

where $\theta_{j}$ denotes the fixed directional angle of the *j*th sensor. The fault point azimuth *θ*_fault_ is inferred using a weighted arithmetic mean based on microphone directional angles, as follows:

(11) $$\theta_{\mathrm{fault}}=\frac{\sum_{j=1}^{N}\omega_{j}c_{j}}{\sum_{j=1}^{N}\omega_{j}}=\frac{\sum_{j=1}^{N}I_{j}^{2}\theta_{j}}{\sum_{j=1}^{N}I_{j}^{2}}$$

### 3.3. Fault Point Search Navigation Based on Zero-Order T–S FLC

#### 3.3.1. Input Variable Membership Functions

(1) Membership Functions for Fault Point Direction

**Figure 6:**
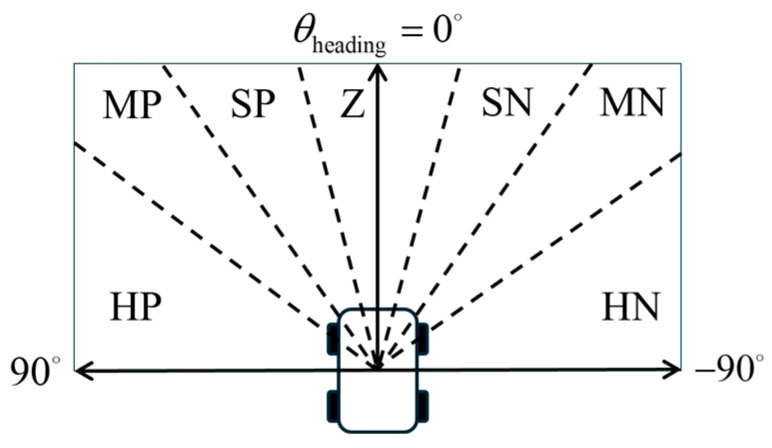
Semantic regions of fault point direction.

The fault point azimuth *θ*_fault_ is used as an input variable for the navigation function. Considering that the robot primarily moves forward, the semantic range of the fuzzy sets is concentrated in the frontal region. Specifically, the 180° frontal sector of the robot is divided into seven fuzzy linguistic sets: Hard-Positive (HP), Med-Positive (MP), Slight-Positive (SP), Zero (Z), Slight-Negative (SN), Med-Negative (MN), and Hard-Negative (HN), as illustrated in Figure 6.

**Figure 7:**
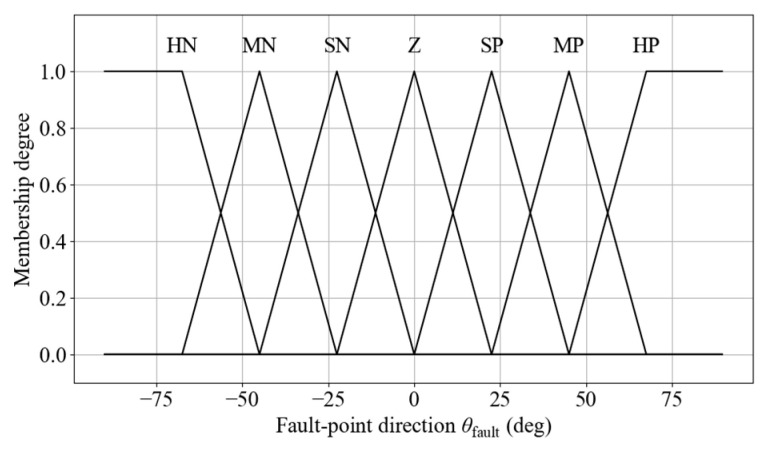
Membership functions for fault point direction.

In the semantic partitioning of the fault point azimuth, the region spanning from L to B is assigned to the PB fuzzy set, while the region from R to BSR is assigned to the NB fuzzy set. Triangular membership functions, along with left-shoulder and right-shoulder functions, are used to model each semantic region. The membership function diagram for the fault point azimuth deviation is shown in Figure 7.

(2) Membership Functions for Distance to the Nearest Equipment Protection Boundary

**Figure 8:**
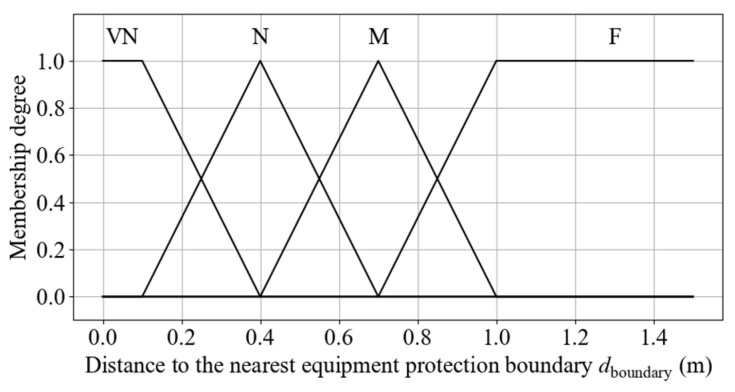
Membership functions for distance to the nearest equipment protection boundary.

To ensure that the diagnostic robot can safely stop before reaching a faulty device during navigation, a physical buffer zone called the equipment protection boundary is defined based on a comprehensive assessment of the maximum driving speed, braking performance, and system delay margins. In this study, it is set to 0.3 m. The distance *d*_boundary_ between the robot and the nearest equipment protection boundary is introduced as an input to the navigation FLC. This distance is defined as the Euclidean distance from the robot’s center of mass to the equipment protection boundary. It is divided into four fuzzy linguistic sets: Far (F), Medium (M), Near (N), and Very Near (VN). Triangular, left-shoulder, and right-shoulder membership functions are used to model these sets. The corresponding membership function diagram is shown in Figure 8.

(3) Membership Functions for Azimuth Difference between the Nearest Equipment and Fault Point

**Figure 9:**
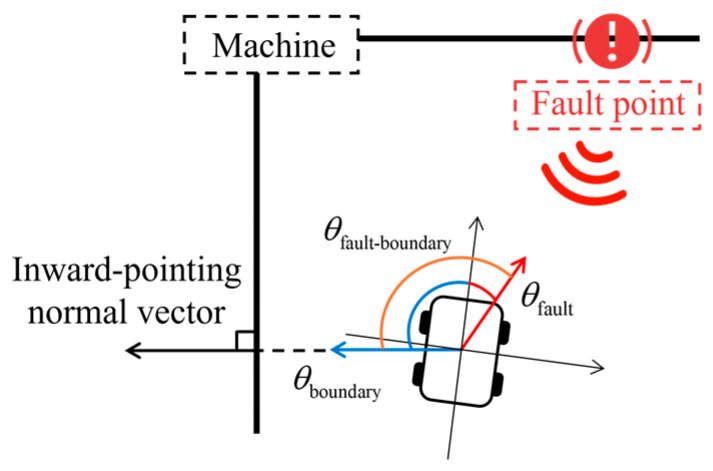
Azimuth difference between the nearest equipment and fault point.

During movement toward the fault point, the diagnostic robot may travel along the equipment due to the positioning of initial inspection points. However, if only the distance to surrounding equipment is considered, the robot may initiate braking when too close to the equipment boundary, which could prematurely terminate the inspection process. In addition, if the fault point is located on the nearest equipment, the robot may begin turning too early during the approach phase, which can reduce the accuracy and effectiveness of reaching the fault point. To address the above issues, the azimuth difference between the nearest equipment and fault point, denoted as Δ*θ*_fault-boundary_, is introduced and defined as follows:

(12) $$\Delta \theta_{\mathrm{fault}-\mathrm{boundary}}={\mathrm{wrap}}_{\deg}\left(\theta_{\mathrm{fault}}-\theta_{\mathrm{boundary}}\right)$$

(13) $${\mathrm{wrap}}_{\deg}(\theta )=(\theta +180^{\circ })\mathrm{mod}360^{\circ }-180^{\circ }$$

where *θ*_boundary_ denotes the azimuth of the nearest boundary equipment which is the inward-facing normal direction of the nearest boundary equipment. The structure of Δ*θ*_fault-boundary_ is illustrated in Figure 9.

When Δ*θ*_fault-boundary_ exceeds 90°, it indicates that the fault point is not located in the nearest boundary equipment. In this case, as the robot moves toward the fault point, it will naturally diverge from the equipment boundary, and braking control is not required. Navigation can therefore proceed uninterrupted.

When Δ*θ*_fault-boundary_ is less than 90° but still relatively large, it indicates that the fault point may be located in the nearest equipment, but some distance remains. At this stage, early turning should be suppressed to allow the robot to continue traveling along with the equipment. Once Δ*θ*_fault-boundary_ becomes smaller, turning can then be initiated, enabling the robot’s heading to approach a perpendicular orientation relative to the equipment surface. This facilitates more accurate alignment of the robot’s front end with faulty equipment, thereby improving the effectiveness of subsequent inspection operations by the manipulator.

**Figure 10:**
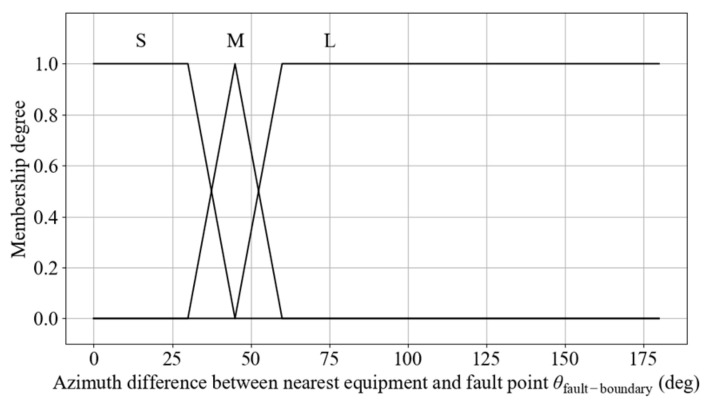
Membership functions for azimuth difference between the nearest equipment and fault point.

The variable Δ*θ*_fault-boundary_ is divided into three fuzzy linguistic sets: Small (S), Medium (M), and Large (L). Triangular, left-shoulder, and right-shoulder membership functions are used to model these sets. The corresponding membership function diagram is shown in Figure 10.

#### 3.3.2. Fuzzy Rule Base Construction

(1) Steering Angle Output Rule Base Construction

The inference of the navigation steering angle output is based on the fault point direction and azimuth difference between the nearest equipment and fault point. The main design principles of the rule base are as follows:

1. The fault point azimuth deviation directly determines the steering angle. Larger deviations correspond to greater steering outputs.
2. The steering angle is limited based on the semantic value of Δ*θ*_fault-boundary_. When the value is large, low-amplitude steering is applied. When the value is small, rapid and full-range steering responses are permitted.

A zero-order T–S fuzzy rule base structure is utilized, in which the consequent of each rule is a fixed constant. The steering angle output is divided into six discrete levels, labeled L1 to L6, where a higher suffix indicates a larger steering angle. Positive and negative signs denote left and right turns, respectively. The rule base for steering angle output in fault point search navigation is shown in Table 1. The specific output values can be determined based on expert knowledge or through optimization algorithms.

(2) Traction Acceleration Output Rule Base Construction

The inference of navigation-traction-acceleration output is based on the fault point direction, azimuth difference between the nearest equipment and fault point, and the distance to the nearest equipment protection boundary. The main design principles of the rule base are as follows:

1. A higher traction force is applied when the robot is far from the equipment boundary, and the output is reduced as it approaches the boundary.
2. When Δ*θ*_fault-boundary_ is large, the system prioritizes maintaining speed. When it is small, the traction force output reverts to being determined by the distance to the equipment boundary.
3. Minor adjustments to the traction force are made based on variations in the fault point azimuth to preserve the robot’s ability to perform necessary directional corrections.

A zero-order T–S fuzzy rule base structure is utilized, in which the consequent of each rule is a fixed constant. The traction force output is divided into forward traction and braking traction. Forward traction is labeled as positive (P) and is subdivided into six levels, P1 to P6. Braking traction is labeled as negative (N) and is subdivided into four levels, N1 to N4. A larger suffix number indicates a greater output magnitude. The traction force rule base is presented in Table 2.

#### 3.3.3. Zero-Order T–S Defuzzification

After the fuzzification of input variables and the execution of fuzzy rule inference, the fuzzy output generated by the controller must be converted into a specific control value to drive the diagnostic robot in performing navigation tasks. A zero-order T–S fuzzy system is employed for defuzzification.

Each rule in the rule base produces a fixed constant as its output. The structure of the *i*th rule is as follows:

(14) $$R_{i}:\mathrm{If}\;x_{1}\;\mathrm{is}\;A_{1}^{i},x_{2}\;\mathrm{is}\;A_{2}^{i},\ldots ,\;x_{n}\;\mathrm{is}\;A_{n}^{i},\;\mathrm{then}\;y_{i}=c_{i}$$

where *x*_1_, *x*_2_, …, *x_n_* denote the input variables; $A_{j}^{i}$ denotes the membership degree of the *j*th input variable in the fuzzy set of the *i*th rule; and *c_i_* is the constant output. The system output *y* is obtained by calculating the weighted average of all activated rules, as follows:

(15) $$y=\frac{\sum_{i=1}^{M}w_{i}\cdot c_{i}}{\sum_{i=1}^{M}w_{i}}$$

where *M* denotes the number of activated fuzzy rules, and *w_i_* denotes the activation strength of the *i*th rule, which is computed by aggregating the membership degrees of all input variables in the rule’s antecedent:

(16) $$w_{i}=\prod_{j=1}^{n}\mu_{A_{j}^{i}}(x_{j})$$

where $\mu_{A_{j}^{i}}$ denotes the membership degree of input variable *x_j_* in the fuzzy set $A_{j}^{i}$.

## 4. Obstacle Avoidance Based on Zero-Order T–S FLC

### 4.1. Static Obstacle Avoidance FLC

**Figure 11:**
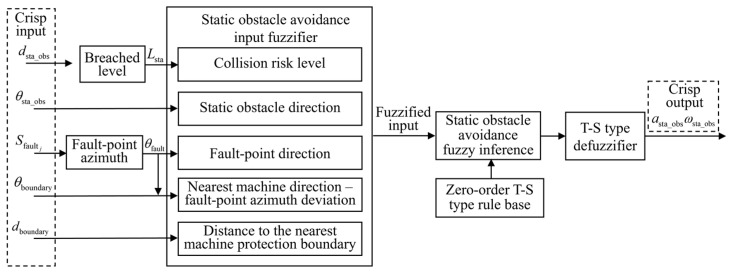
FLC process for static obstacle avoidance.

A zero-order T–S FLC method is utilized, with the control process illustrated in Figure 11. The system first acquires scene information, including the distance to the static obstacle *d*_sta_obs_ and the azimuth of the static obstacle *θ*_sta_obs_. The static obstacle distance is preprocessed to determine the breached level. The input for the navigation section is also utilized. The input variables are then fuzzified, followed by fuzzy inference based on the rule base. Finally, defuzzification is performed to generate traction acceleration *a*_sta_obs_ and front-wheel steering angle $\omega_{\mathrm{sta}\_\mathrm{obs}}$.

#### 4.1.1. Input Variable Membership Functions

To enable effective avoidance of static obstacles, the distance and direction of static obstacles are considered.

(1) Membership Functions for Static Obstacle Breached Level

Conventional static obstacle avoidance strategies typically use the Euclidean distance between the robot and the obstacle as a fuzzy control input. However, this approach does not explicitly reflect braking performance, sensor uncertainty, or other sources of variability, which limits the ability to respond effectively to potential risks. To address this limitation, a safety boundary is defined around the robot, and a breached level is introduced to evaluate the collision risk posed by static obstacles. The static obstacle breached level *L*_sta_ is defined as follows:

(17) $$L_{\mathrm{sta}}=\frac{d_{\mathrm{sta}\_\mathrm{obs}}}{R_{\mathrm{safe}\_\mathrm{sta}}}$$

where *d*_sta_obs_ denotes the actual distance between the robot and the obstacle, and *R*_safe_sta_ denotes the radius of the safety boundary. The calculation methods for both are as follows:

(18) $$d_{\mathrm{sta}\_\mathrm{obs}}=\sqrt{{\left(x_{\mathrm{sta}\_\mathrm{obs}}-x_{\mathrm{robot}}\right)}^{2}+{\left(y_{\mathrm{sta}\_\mathrm{obs}}-y_{\mathrm{robot}}\right)}^{2}}$$

(19) $$R_{\mathrm{safe}\_\mathrm{sta}}=d_{\mathrm{stop}}+d_{\mathrm{delay}}+\varepsilon_{\mathrm{sensor}}$$

(20) $$d_{\mathrm{stop}}=\frac{v_{\max}^{2}}{2a_{\max}}$$

(21) $$d_{\mathrm{delay}}=v_{\max}t_{\mathrm{delay}}$$

where (*x*_sta_obs_, *y*_sta_obs_) and (*x*_robot_, *y*_robot_) denote the positions of the static obstacle and the robot, respectively. *d*_stop_ and *d*_delay_ denote the braking distance and delay margin. *v*_max_ and *a*_max_ are the maximum configured linear speed and acceleration of the robot. *t*_delay_ refers to the total delay time across perception, decision-making, and execution stages, and $\varepsilon_{\mathrm{sensor}}$ the sensor error.

**Figure 12:**
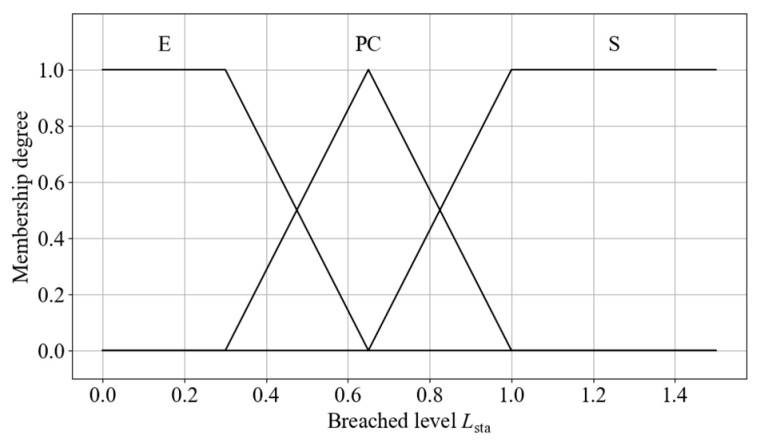
Membership functions for breached level.

The breached level is divided into three fuzzy linguistic sets: Emergency (E), Possible Collision (PC), and Safe (S). When *L*_sta_ > 1, it falls within the S category. When *L*_sta_ < 1, it indicates that the obstacle has entered the safety boundary, prompting activation of avoidance strategies based on the PC and E. The E category denotes a critical situation that requires more aggressive avoidance behavior. This normalized representation allows the membership function shapes and intervals to be reused across different scenarios and platforms. The designed input membership functions are shown in Figure 12.

(2) Membership Functions for Obstacle Azimuth

The spatial orientation of an obstacle plays a critical role in determining the avoidance path. The static obstacle azimuth is calculated as follows:

(22) $$\varphi_{\mathrm{sta}\_\mathrm{obs}}=\mathrm{wrap}\left(\mathrm{atan}2(y_{\mathrm{sta}\_\mathrm{obs}}-y_{\mathrm{robot}},x_{\mathrm{sta}\_\mathrm{obs}}-x_{\mathrm{robot}})-\varphi_{\mathrm{robot}}\right)$$

(23) wrap(φ)=(φ+π)mod2π−π

where $\varphi_{\mathrm{sta}\_\mathrm{obs}}$ and $\varphi_{\mathrm{robot}}$ denote the heading angles of the static obstacle and the robot, respectively, expressed in radians in the global coordinate frame. The azimuth angle is then converted into degrees for use in membership degree calculation:

(24) $$\theta_{\mathrm{sta}\_\mathrm{obs}}=\varphi_{\mathrm{sta}\_\mathrm{obs}}\times \frac{180}{\pi }$$

**Figure 13:**
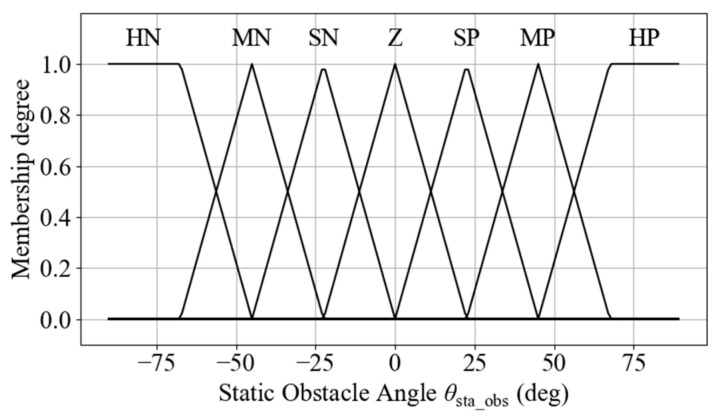
Membership functions for obstacle azimuth.

The static obstacle azimuth is divided into seven fuzzy linguistic sets: Hard-Positive (HP), Med-Positive (MP), Slight-Positive (SP), Zero (Z), Slight-Negative (SN), Med-Negative (MN), and Hard-Negative (HN). These spatial semantic regions are consistent with the partitioning used for fault point direction. Triangular, left-shoulder, and right-shoulder membership functions are used to construct fuzzy sets for obstacle azimuth. The membership function diagram is shown in Figure 13.

#### 4.1.2. Fuzzy Rule Base Construction

(1) Steering Angle Output Rule Base Construction

The inference of the static obstacle avoidance steering angle output is based on static obstacle azimuth, fault point direction, and the distance to the nearest equipment protection boundary. The main design principles of the rule base are as follows:

1. The required steering direction and magnitude are determined based on the obstacle azimuth. The steering direction is opposite to the obstacle’s orientation, and as the obstacle azimuth increases, the required steering angle magnitude decreases.
2. When the obstacle is located directly in front of the robot, steering is directed toward the fault point direction.
3. When the distance to the nearest equipment is small, the avoidance direction is selected to move away from the equipment during the obstacle avoidance process.

Same as the navigation function, a zero-order T–S fuzzy rule base structure is utilized. The steering angle output is divided into three levels, labeled L1 to L3. When the breached level is classified as S-level, no steering output is applied. For the PC-level, the steering angle outputs are specified as shown in Table 3. For the E-level, the output value is enhanced based on the PC-level.

(2) Traction Acceleration Output Rule Base Construction

The inference of the static obstacle-avoidance traction-acceleration output is based on the breached level, static obstacle azimuth, the distance to the nearest equipment protection boundary, and azimuth difference between the nearest equipment and fault point. The main design principles of the rule base are as follows:

1. Traction acceleration is reduced as the breached level decreases.
2. As the obstacle orientation deviates further from the front toward the sides, its impact on navigation decreases. In such cases, the traction acceleration is moderately increased.
3. When the robot is close to equipment, the traction acceleration is reduced.

A zero-order T–S fuzzy rule base structure is utilized. Forward traction acceleration is divided into five levels, labeled P1 to P5, while braking output is represented by a single level, N1. When the breached level is classified as S-level, the traction acceleration output is set to P5. For the PC-level, traction acceleration outputs are defined as shown in Table 4. For the E-level, the forward traction value is reduced, and the braking output value is increased based on PC-level.

### 4.2. Dynamic Obstacle Avoidance FLC

**Figure 14:**
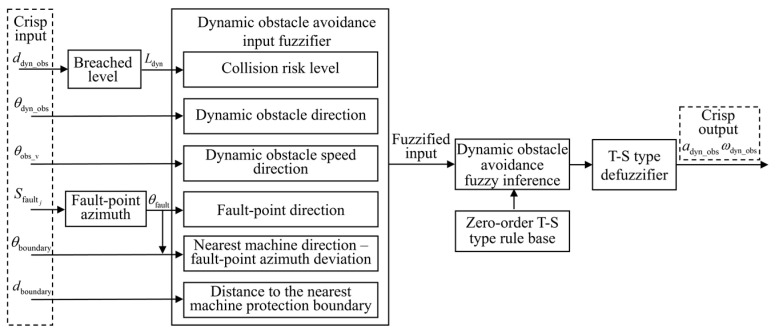
FLC process for dynamic obstacle avoidance.

A zero-order T–S FLC method is utilized, with the control process illustrated in Figure 14. The system first acquires scene information, including the distance to the dynamic obstacle *d*_dyn_obs_, azimuth of the dynamic obstacle *θ*_dyn_obs_, and speed direction angle of the dynamic obstacle *θ*_v_obs_. The dynamic obstacle distance is preprocessed to determine the breached level. The inputs for the navigation section are also utilized. The input variables are then fuzzified, followed by fuzzy inference based on the rule base. Finally, defuzzification is performed to generate traction acceleration *a*_dyn_obs_ and front-wheel steering angle $\omega_{\mathrm{dyn}\_\mathrm{obs}}$.

#### 4.2.1. Input Variable Membership Functions

To enhance the risk identification and response capability of the obstacle avoidance control system in dynamic environments, obstacle speed direction is additionally introduced. Specifically, the fuzzy control inputs of the dynamic obstacle avoidance module consist of three components: breached level, obstacle azimuth, and obstacle speed direction.

(1) Membership Functions for Dynamic Obstacle Breached Level

To more effectively capture the potential collision risk posed by rapidly approaching dynamic obstacles, a dynamic safety boundary is constructed based on the relative speed, extending the static obstacle safety boundary. The radius of the dynamic safety boundary, *R*_safe_dyn_, is calculated as follows:

(25) $$R_{\mathrm{safe}\_\mathrm{dyn}}=R_{\mathrm{sta}}+k\times \frac{v_{\mathrm{rel}}^{2}}{2a_{\max}}$$

(26) $$v_{\mathrm{rel}}=\left\|{\vec{v}}_{\mathrm{obs}}-{\vec{v}}_{\mathrm{robot}}\right\|$$

where *R*_sta_ denotes the radius of the static safety boundary; *v*_rel_ is the magnitude of the relative speed between the robot and the dynamic obstacle, ${\vec{v}}_{\mathrm{obs}}$ and ${\vec{v}}_{\mathrm{robot}}$ are the speed vectors of the obstacle and the robot, respectively; and *k* is a time-dimensional scaling factor that linearly converts relative velocity into distance, representing the minimum time-to-collision margin. In this study, *k* is set to 2 s, with larger values triggering earlier avoidance and providing greater safety margins. The distance between the robot and the dynamic obstacle is computed in the same manner as for static obstacles. The breached level for dynamic obstacles, *L*_dyn_, is calculated as follows:

(27) $$L_{\mathrm{dyn}}=\frac{d_{\mathrm{dyn}\_\mathrm{obs}}}{R_{\mathrm{safe}\_\mathrm{dyn}}}$$

Due to the normalized formulation of the breached level, the semantic categories for static and dynamic obstacles are identical. As a result, the same membership functions for breached level used in static obstacle avoidance are applied in the dynamic obstacle avoidance fuzzy inference process, thereby simplifying the design of both the controller and the rule base.

(2) Membership Functions for Dynamic Obstacle Azimuth

**Figure 15:**
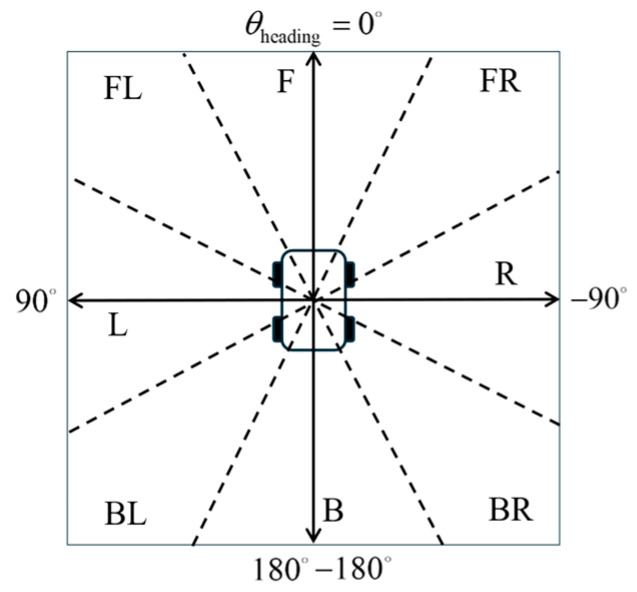
Semantic regions of the dynamic obstacle azimuth.

The calculation of the dynamic obstacle azimuth *θ*_dyn_obs_ is identical to that used for static obstacle azimuth. Since dynamic obstacles may approach from any direction around the robot, the dynamic obstacle azimuth is divided into seven fuzzy linguistic sets: Front (F), Front-Left (FL), Left (L), Back-Left (BL), Back (B), Back-Right (BR), Right (R), and Front-Right (FR). Each semantic label covers a 45° angular sector. The semantic partitioning is illustrated in Figure 15.

**Figure 16:**
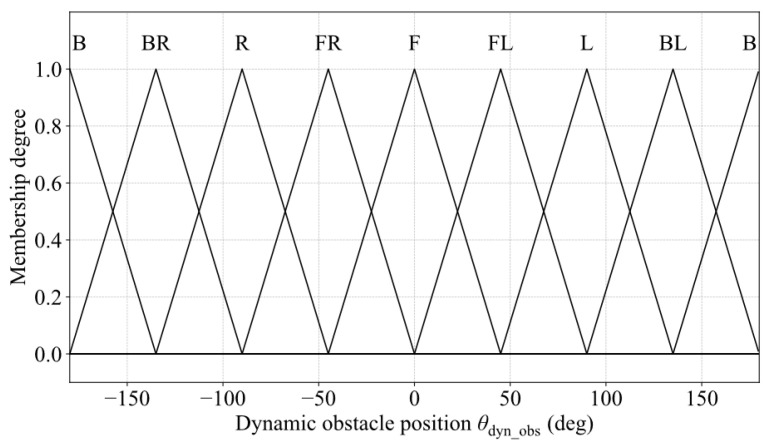
Membership functions for dynamic obstacle azimuth.

Triangular membership functions are used to construct fuzzy sets for the dynamic obstacle azimuth. The corresponding membership function diagram is shown in Figure 16.

(3) Membership Functions for Dynamic Obstacle Speed Direction

To enhance the understanding of dynamic obstacle motion trends, the speed direction angle of the obstacle is introduced. It is calculated as follows:

(28) $$\varphi_{\mathrm{obs}\_v}=\mathrm{wrap}\left(\mathrm{atan}2\left({\dot{y}}_{\mathrm{obs}},{\dot{x}}_{\mathrm{obs}}\right)-\varphi_{\mathrm{robot}}\right)$$

where ${\dot{x}}_{\mathrm{obs}}$ and ${\dot{y}}_{\mathrm{obs}}$ denote the lateral and longitudinal speed components of the obstacle in the global coordinate frame, respectively. The resulting angle is then converted into degrees to serve as the input for the membership function:

(29) $$\theta_{\mathrm{obs}\_v}=\varphi_{\mathrm{obs}\_v}\times \frac{180}{\pi }$$

**Figure 17:**
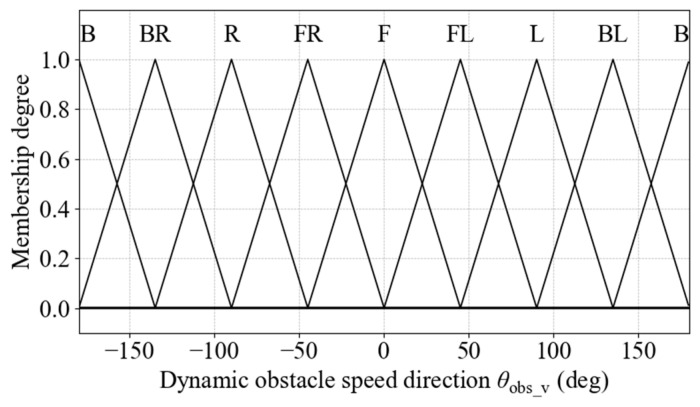
Membership functions for dynamic obstacle speed direction.

Dynamic obstacle speed direction is divided into seven fuzzy linguistic sets: Front (F), Front-Left (FL), Left (L), Back-Left (BL), Back (B), Back-Right (BR), Right (R), and Front-Right (FR). The semantic partitioning is identical to that used for the dynamic obstacle azimuth, and triangular membership functions are employed to construct the corresponding fuzzy sets, as illustrated in Figure 17.

#### 4.2.2. Fuzzy Rule Base Construction

(1) Steering Angle Output Rule Base Construction

The inference of the dynamic obstacle avoidance steering-angle-output is based on breached level, dynamic obstacle azimuth, dynamic obstacle speed direction, and the distance to the nearest equipment protection boundary. The main design principles of the rule base are as follows:

1. The steering strategy is selected to be perpendicular to the obstacle speed direction.
2. When the relative motion indicates that the obstacle is moving away, and both are traveling in approximately parallel directions, no steering action is applied.
3. When the distance to the nearest equipment is small, the avoidance direction is selected to move away from the equipment during the obstacle avoidance process.

Same as the static obstacle avoidance function, a zero-order T–S fuzzy rule base structure is utilized. The steering angle output is divided into three levels, labeled L1 to L3. When the breached level is classified as S-level, no steering output is applied. For the PC-level, the steering angle outputs are specified as shown in Table 5. For the E-level, the output value is enhanced based on the PC-level.

The obstacle speed direction influences the magnitude of the steering angle during equipment protection. The rule design for equipment protection in dynamic obstacle avoidance is presented in Table 6.

(2) Traction Acceleration Output Rule Base Construction

The inference of the dynamic obstacle avoidance traction acceleration output is based on breached level, dynamic obstacle azimuth, dynamic obstacle speed direction, and the distance to the nearest equipment protection boundary. The main design principles of the rule base are as follows:

1. When the motion trend is directed toward the front of the robot, a moderate reduction is applied. When the motion is directed toward the robot, a significant reduction is applied. If the obstacle is moving away, the normal traction output is maintained.
2. For extreme left or right obstacle speed directions, braking is applied.
3. Based on the severity of the breached level, forward traction force is reduced while braking traction is increased.

A zero-order T–S fuzzy rule base structure is utilized. Forward traction acceleration is divided into three levels, labeled P1 to P3, while braking output is represented by a single level, N1. When the breached level is classified as S-level, the traction acceleration output is set to P5. For the PC-level, traction acceleration outputs are defined as shown in Table 7. For the E-level, the forward traction value is reduced, and the braking output value is increased based on PC-level.

When the distance to the nearest equipment protection boundary falls within the N or VN categories, P2 is applied under the PC-level, and P1 is applied under the E-level.

## 5. Function Switching Strategy Based on Mamdani-Type FIS

### 5.1. Function Coordination Between Navigation and Obstacle Avoidance

To enable switching between navigation and obstacle avoidance functions under varying environmental demands, obstacle avoidance necessity is inferred using a Mamdani-type FIS. The inference is based on two key considerations: the degree of impact on navigation and the safety of the robot.

**Figure 18:**
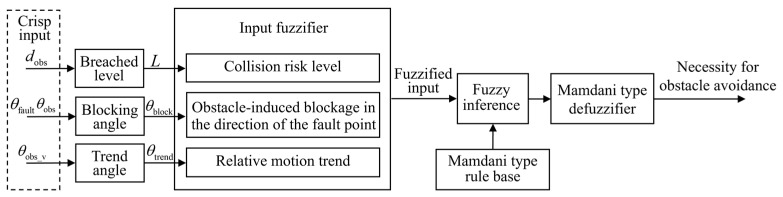
FIS process for obstacle avoidance necessity.

The inference process is illustrated in Figure 18. The system first performs preprocessing and feature extraction on the raw input data. The breached level *L* is calculated based on the obstacle distance *d*_obs_; the block angle *θ*_block_ is derived from the deviation between the fault point azimuth *θ*_fault_ and the obstacle azimuth *θ*_obs_; the motion trend angle *θ*_trend_ is computed based on the obstacle speed direction *θ*_obs_v_. The input variables are then fuzzified and processed using a Mamdani-type fuzzy rule base. Defuzzification is performed to obtain obstacle avoidance necessity.

The degree of impact on navigation is evaluated using the angular difference between the obstacle azimuth and the fault point azimuth, defined as the blocking angle *θ*_block_. It is calculated as follows:

(30) $$\theta_{\mathrm{block}}={\mathrm{wrap}}_{\deg}\left(\theta_{\mathrm{fault}}-\theta_{\mathrm{sta}\_\mathrm{obs}}\right)\;or{\;\mathrm{wrap}}_{\deg}\left(\theta_{\mathrm{fault}}-\theta_{\mathrm{dyn}\_\mathrm{obs}}\right)$$

**Figure 19:**
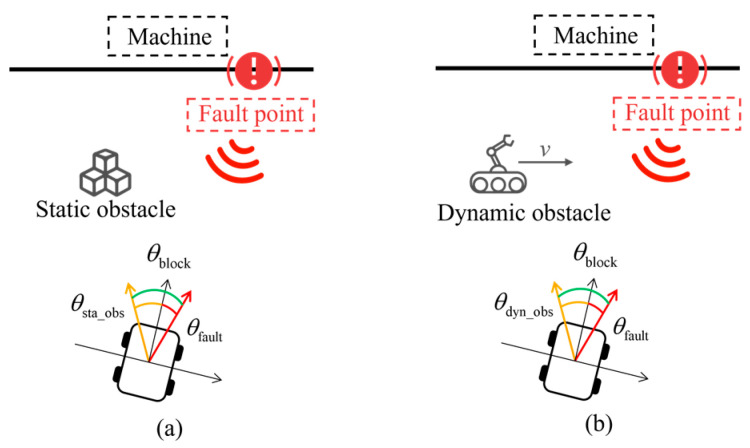
Composition of blocking angle. (**a**) Angular configurations in static scenarios. (**b**) Angular configurations in dynamic scenarios.

The structure of the blocking angle *θ*_block_ is illustrated in Figure 19, where (a) and (b) denote the angular configurations in static and dynamic obstacle scenarios, respectively. When the angular difference is zero, the fault point is fully blocked by the obstacle, and avoidance is required for the robot to reach the target.

**Figure 20:**
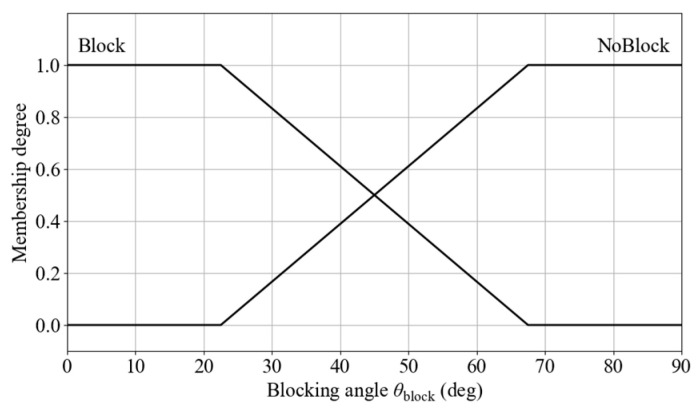
Membership functions for blocking angle.

The membership function for blocking degree includes two semantic categories: Block and NoBlock, with 45° defined as the intersection point between them. The membership function diagram is shown in Figure 20. The input is defined as $x_{1}\;=\;\theta_{\mathrm{block}\;}\in \;\{-180{}^{\circ},\;180{}^{\circ}\}$. The fuzzy set is defined as $\mu_{i}^{\left(1\right)}(x_{1})$, where i ∈ {Block, NoBlock}.

The two core elements of safety are the motion trend of the obstacle and the breached level. These are characterized by two input variables: trend angle and breached level. The membership function for breached level is consistent with that used in obstacle avoidance. The breached level input is defined as follows:

(31) $$x_{2}=D_{\mathrm{breached}}\in \{1,\;1.5\}$$

(32) $$D_{\mathrm{breached}}=\left\{\begin{array}{cc} D_{b\_\mathrm{dyn}}, & \mathrm{Dynamic}\;\mathrm{obstacle}\;\mathrm{avoidance}\\ D_{b\_\mathrm{sta}}, & \mathrm{Static}\;\mathrm{obstacle}\;\mathrm{avoidance} \end{array}\right.$$

The fuzzy set is defined as $\mu_{j}^{\left(2\right)}(x_{2})$, where j ∈ {S, PC, E}. To describe the motion trend of an obstacle relative to the robot, a trend angle is introduced to determine whether the obstacle is approaching or receding. The trend angle for a dynamic obstacle is calculated as follows:

(33) $$\theta_{\mathrm{dyn}\_\mathrm{trend}}={\mathrm{wrap}}_{\deg}(\theta_{\mathrm{rel}\_v}-\theta_{obs})$$

where *θ*_rel_v_ is the relative speed direction of the obstacle. For static obstacles, the relative motion in the robot’s local coordinate frame can be interpreted as movement in the direction opposite to the robot’s heading. The trend angle for static obstacles is calculated as follows:

(34) $$\theta_{\mathrm{sta}\_\mathrm{trend}}={\mathrm{wrap}}_{\deg}(-180^{\circ }-\theta_{obs})$$

The input is defined as:

(35) $$x_{3}=\theta_{\mathrm{trend}}\in [-180^{\circ },180^{\circ }]$$

(36) $$\theta_{\mathrm{trend}}=\left\{\begin{array}{cc} \theta_{\mathrm{dyn}\_\mathrm{trend}}, & \mathrm{Dynamic}\;\mathrm{obstacle}\;\mathrm{avoidance}\\ \theta_{\mathrm{sta}\_\mathrm{trend}}, & \mathrm{Static}\;\mathrm{obstacle}\;\mathrm{avoidance} \end{array}\right.$$

**Figure 21:**
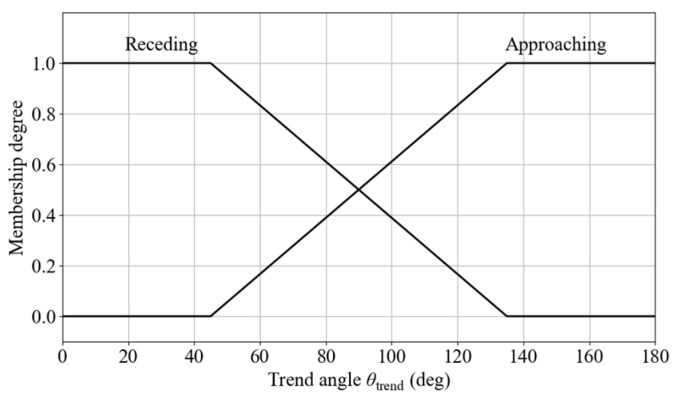
Membership functions for trend angle.

Two fuzzy linguistic sets are defined to describe the obstacle trend: approaching and receding. The fuzzy set is defined as $\mu_{k}^{\left(3\right)}(x_{3})$, where k ∈ {Approaching, Receding}. The membership functions are shown in Figure 21.

**Figure 22:**
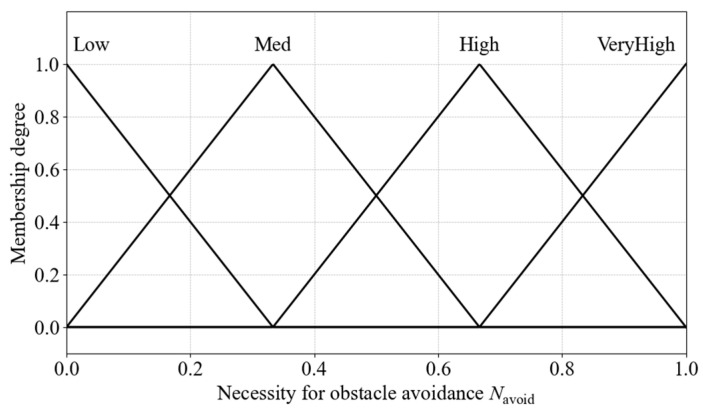
Membership functions for obstacle avoidance necessity.

Obstacle avoidance necessity is divided into seven fuzzy linguistic sets: Low, Med, High, and VeryHigh. The output membership functions are shown in Figure 22.

Based on the input and output membership functions, the fuzzy rule base for obstacle avoidance necessity is constructed, as shown in Table 8.

Mamdani-type fuzzy inference computes rule activation strength based on the minimal intersection applied to IF–THEN rules. Rules are defined as follows:

(37) $$R_{r}:\mathrm{IF}\;x_{1}\;\mathrm{is}\;A_{i}\;\mathrm{AND}\;x_{2}\;\mathrm{is}\;B_{j}\;\mathrm{AND}\;x_{3}\;\mathrm{is}\;C_{k}\;\mathrm{THEN}\;y\;\mathrm{is}\;D_{r}$$

Rule activation strength *α_r_* is calculated as follows:

(38) $$\alpha_{r}=\min\left(\mu_{i}^{\left(1\right)}(x_{1}),\mu_{j}^{\left(2\right)}(x_{2}),\mu_{k}^{\left(3\right)}(x_{3})\right)$$

When a single input in the rule antecedent corresponds to multiple fuzzy labels, the maximum membership degree among those labels is used for that input. The output label is defined as follows:

(39) $$D_{r}\in \{\mathrm{Low},\mathrm{Med},\mathrm{High},\mathrm{VeryHigh}\}$$

The results of all rules are aggregated according to their output linguistic label. For each output label, the final aggregated membership value is determined as the maximum activation strength among all rules concluding with that label:

(40) $$\mu_{D}(y)=\max\left(\alpha_{r}|D_{r}=D\right),\;D\in D$$

where D is the output linguistic label set. After aggregation, the membership function of the output fuzzy set is formed as follows:

(41) $$\mu_{\mathrm{out}}(y)=\underset{D\in D}{\cup }\left[\mu_{D}(y)\cdot \chi_{D}(y)\right]$$

where $\chi_{D}(y)$ denotes the membership function for label *D*.

The output fuzzy set is defuzzified using the centroid method to obtain the final output value $\mu_{\mathrm{avoid}}$:

(42) $$\mu_{\mathrm{avoid}}=\frac{\int_{Y}y\cdot \mu_{\mathrm{out}}(y)dy}{\int_{Y}\mu_{\mathrm{out}}(y)dy}$$

The final output of the control system ($a_{\mathrm{control}}$, $\omega_{\mathrm{control}}$) is calculated as follows:

(43) $$(a_{\mathrm{control}},\omega_{\mathrm{control}})=\left\{\begin{array}{cc} (a_{\mathrm{obs}},\omega_{\mathrm{obs}}), & \mathrm{if}\;\mu_{\mathrm{avoid}}\geq T_{\mathrm{avoid}}\\ (a_{\mathrm{nav}},\omega_{\mathrm{nav}}), & \mathrm{otherwise} \end{array}\right.$$

where ($a_{\mathrm{obs}}$, $\omega_{\mathrm{obs}}$) and ($a_{\mathrm{nav}}$, $\omega_{\mathrm{nav}}$) are the output of the obstacle avoidance function and navigation function, which are used to control the robot; *T*_avoid_ denotes the obstacle avoidance threshold, which is set to 0.5 in this study based on the semantic median partition point.

### 5.2. Selection of Priority Obstacle for Avoidance

**Figure 23:**
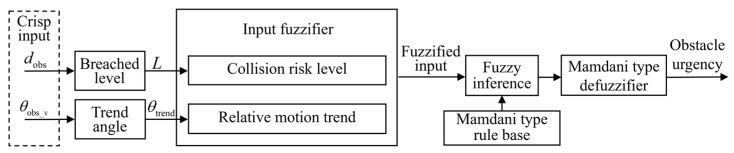
FIS process for obstacle urgency.

In multi-obstacle scenarios, it is necessary to identify the most critical obstacle for avoidance, considering both the distance and motion trend of each obstacle. Based on the obstacle distance *d*_obs_ and speed direction *θ*_obs_v_, the breached level and trend angle are computed and fuzzified. Mamdani-type FIS is then applied to infer the avoidance urgency for each obstacle. The inference process is illustrated in Figure 23.

**Figure 24:**
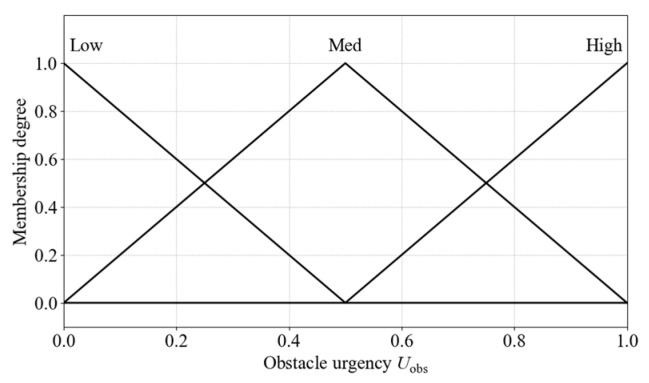
Membership functions for obstacle urgency.

The definitions and membership functions of breached level and trend angle are identical to those used in the obstacle avoidance necessity inference. Obstacle urgency is divided into three fuzzy linguistic sets: Low, Med, and High. The corresponding output membership functions are shown in Figure 24.

The urgency of each obstacle is evaluated using the same inference method as that applied for obstacle avoidance necessity. The fuzzy rule base for inferring the avoidance urgency of each obstacle is presented in Table 9.

## 6. Simulation Test

**Figure 25:**
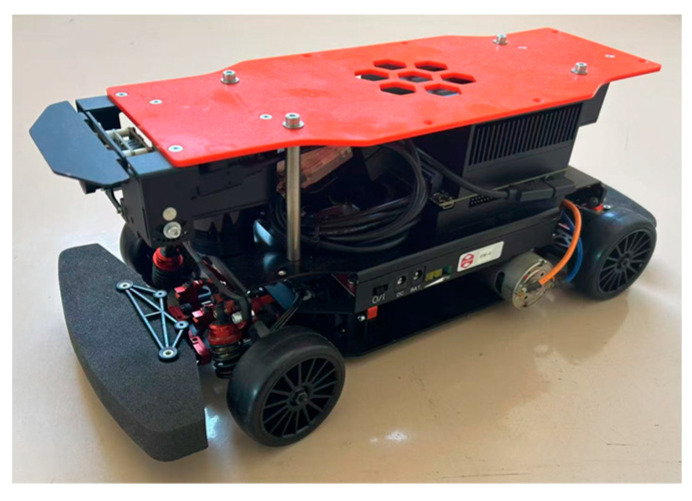
RoboCar 1/10.

To validate the proposed FLC method, simulation scenarios are designed according to specific functional modules, including fault point search navigation, static and dynamic obstacle avoidance, and multi-objective planning. The model of the diagnostic robot used in the simulation is based on the RoboCar 1/10 developed by ZMP Corporation, Tokyo, Japan, as shown in Figure 25. The corresponding parameters are listed in Table 10. The test environment simulates a factory setting, with each inspection area covering 3 to 5 m^2^. Due to the limited space, the desired speed for search and navigation tasks is set to 0.1 m/s. Larger braking accelerations in the rule base are required to support higher rated speeds, and the present rule-based design can accommodate an increase of approximately 16% above the rated speed.

In the fault point search navigation stage, the core functions of the inspection robot include fault point navigation, obstacle avoidance, and collision-free operation with boundary equipment. In the simulations, these functions were evaluated, respectively, by the distance from the robot’s front center to the fault point, the minimum distance between the robot and obstacles, and the minimum distance between the robot and boundary equipment.

### 6.1. Fault Point Search Navigation Validation

**Figure 26:**
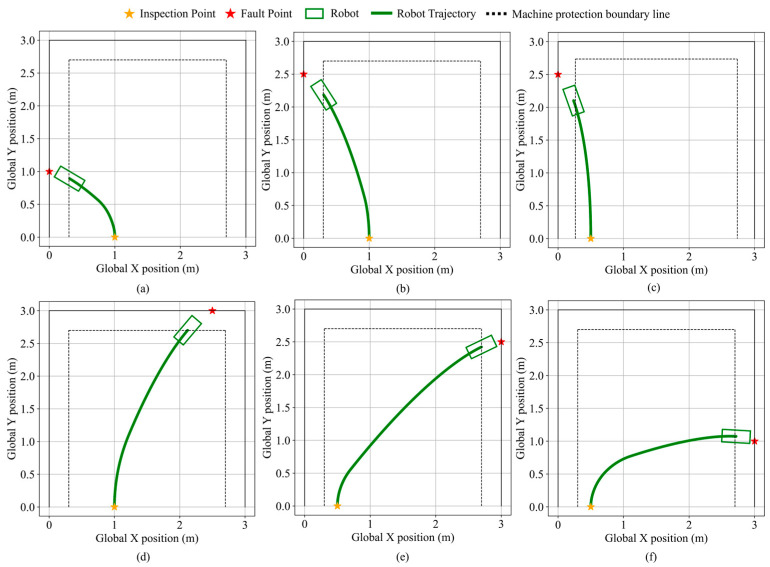
Robot state during fault point search navigation validation. (**a**) Close-Range navigation validation. (**b**) Right-boundary navigation validation. (**c**) Boundary-following navigation validation. (**d**) Top-boundary navigation validation. (**e**,**f**) Left-boundary navigation validation.

The simulation validation is conducted in a 3 m square environment. The left, top, and right boundaries are designated as factory equipment, where simulated faults emit fault-related acoustic signals. The diagnostic robot starts from a stationary state at an inspection point located along the bottom edge and performs fault point search and navigation toward randomly generated fault locations in the scene. Robot states during the validation are shown in Figure 26.

A comparison between Figure 26b,c demonstrates that when the fault point is located on the current equipment but at a certain distance, the steering angle output is suppressed based on the angular difference between the nearest equipment orientation and the fault point direction. This suppression enables the robot to exhibit boundary-following behavior. As the angular difference gradually decreases, regular steering is resumed, allowing the robot to continue smooth motion and approach the fault point with an orientation as perpendicular as possible to the equipment surface.

As illustrated in Figure 26f, even when the robot is near the boundary equipment, if the fault point is not in the direction of the currently nearest equipment, the system remains in boundary-following mode. The robot does not perform deceleration but continues to apply traction force, maintaining a stable trajectory toward the fault point and ultimately stopping safely in its vicinity. The performance is presented in Table 11. To further validate the algorithm, a boundary-based simulation is conducted by randomly selecting points along the edges of the scenario. The results indicate that, due to the turning limitations of the robot, the algorithm cannot reach the vicinity of the target point when navigating along the right boundary below (0, 0.668). This limitation is compensated by assigning patrol points in the corresponding region during the preceding patrol path planning step. For other boundary regions with randomly selected points, the results demonstrate that equipment safety is maintained, and the average distance between the robot’s front endpoint and the fault point is 18.7 cm.

### 6.2. Static Obstacle Avoidance Validation

**Figure 27:**
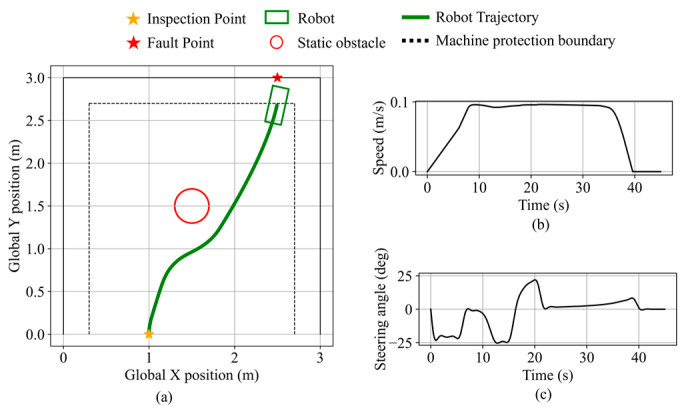
Robot states during static obstacle avoidance in validation scenario 1. (**a**) Robot trajectory. (**b**) Robot speed. (**c**) Robot steering angle.

In validation scenario 1, the inspection and fault points are the same as in Figure 26d. A static circular obstacle with a radius of 0.2 m is placed at (1.5, 1.5). Robot states during the validation are shown in Figure 27. As the obstacle enters the safety boundary and continues to approach, the obstacle avoidance necessity exceeds the threshold, thereby triggering avoidance behavior. The robot performs rightward avoidance while considering the fault point direction, maintaining a minimum obstacle clearance of 12.0 cm. After the obstacle no longer presents a blockage and recedes, the robot resumes navigation and stops along the equipment protection boundary, maintaining a clearance of 9.2 cm from the equipment. The final distance from the robot’s front center to the fault point is 11.9 cm.

**Figure 28:**
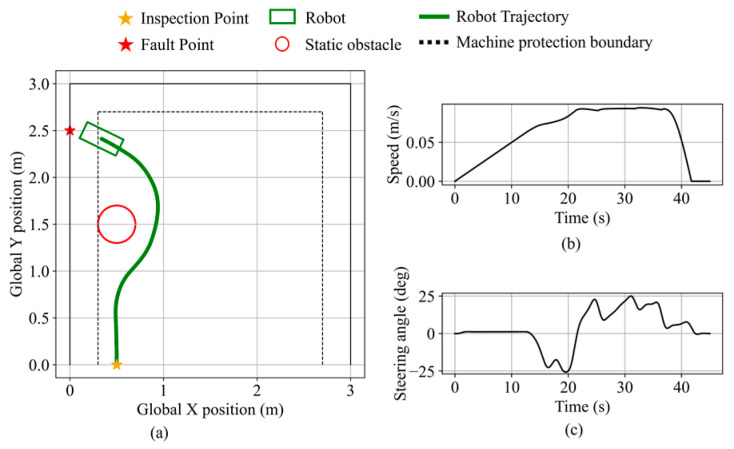
Robot states during static obstacle avoidance in validation scenario 2. (**a**) Robot trajectory. (**b**) Robot speed. (**c**) Robot steering angle.

The obstacle avoidance behavior near boundary equipment is validated in the second validation scenario. The inspection point is set at (0.5, 0.0), the fault point at (0.0, 2.5), and a static obstacle with a 0.2 m radius is placed at (0.5, 1.5). The robot state during the validation is shown in Figure 28. Initially navigating toward the fault point direction, the robot switches to obstacle avoidance mode based on the inferred obstacle avoidance necessity and performs avoidance in the direction opposite to the nearby equipment. The minimum distance to the obstacle is 11.1 cm. The robot then resumes navigation and stops near the fault point along the equipment protection boundary, maintaining a clearance of 10.4 cm from the equipment. The final distance from the robot’s front center to the fault point is 14.6 cm.

**Figure 29:**
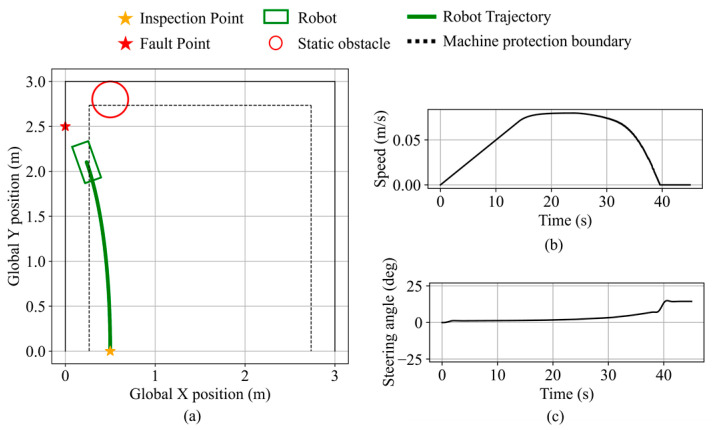
Robot states during static obstacle avoidance in validation scenario 3. (**a**) Robot trajectory. (**b**) Robot speed. (**c**) Robot steering angle.

In validation scenario 3, the inspection and fault points remain the same as in scenario 2, with the obstacle placed at (0.5, 2.8), as shown in Figure 29. Due to the minimal blockage by the obstacle, the obstacle avoidance necessity remains low, and the robot maintains navigation mode. It stops near the fault point along the equipment protection boundary, maintaining a clearance of 7.6 cm from the equipment. The final distance from the robot’s front center to the fault point is 25.6 cm.

### 6.3. Dynamic Obstacle Avoidance Validation

**Figure 30:**
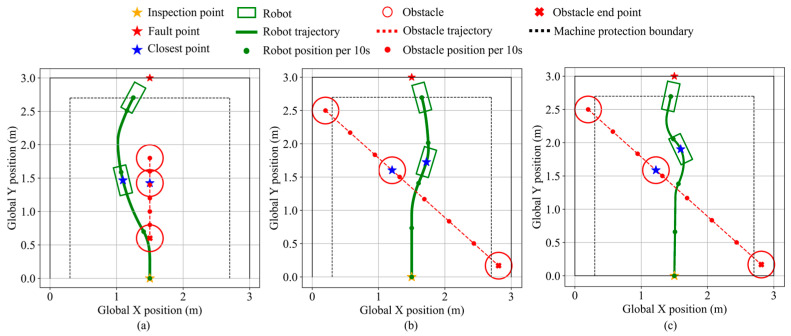
Robot states during dynamic obstacle avoidance in validation scenario 1 and 2. (**a**) Dynamic obstacle approaching head-on. (**b**) Dynamic obstacle approaching from front-left. (**c**) MPC comparison test.

Two dynamic obstacle scenarios are constructed: one approaching head-on and the other from the front-left, with simulation results shown in Figure 30. In the head-on case, the expanded dynamic safety boundary enables early avoidance triggering based on the relative speed. As the fault point remains directly ahead, left and right turns are equivalent; a left turn is selected. The minimum distance to the obstacle is 8.8 cm. The robot then resumes navigation and stops near the fault point, maintaining a clearance of 6.0 cm from the equipment. The final distance from the robot’s front center to the fault point is 18.0 cm. In the front-left approach scenario, the robot turns right to avoid the obstacle, maintaining a minimum distance of 16.8 cm from the obstacle and 7.3 cm from the equipment. The final distance from the robot’s front-center to the fault point is 13.7 cm.

In the same scenario, a Model Predictive Controller (MPC) is designed for comparison using a step size of 0.05, a prediction horizon of 10, and three optimization iterations per step. The comparison shows that the trajectory of the MPC-controlled robot exhibits a certain degree of overshoot. The minimum distance between the MPC-controlled robot and the obstacle is 18.6 cm, the minimum distance to the equipment is 7.1 cm, and the final distance from the robot’s front center to the fault point is 16.5 cm. Under the same hardware configuration on a Lenovo laptop (Lenovo Group Limited, Beijing, China) equipped with an 11th Gen Intel(R) Core(TM) i7-11800H processor (Intel Corporation, Santa Clara, CA, USA) and 32 GB memory, fuzzy logic control requires significantly less computational power, remaining at a constant level of approximately 1.09 ms per step, whereas MPC requires 50.2 ms per step. These results suggest that fuzzy logic control offers greater practical feasibility for inspection robots by avoiding excessive computational costs.

**Figure 31:**
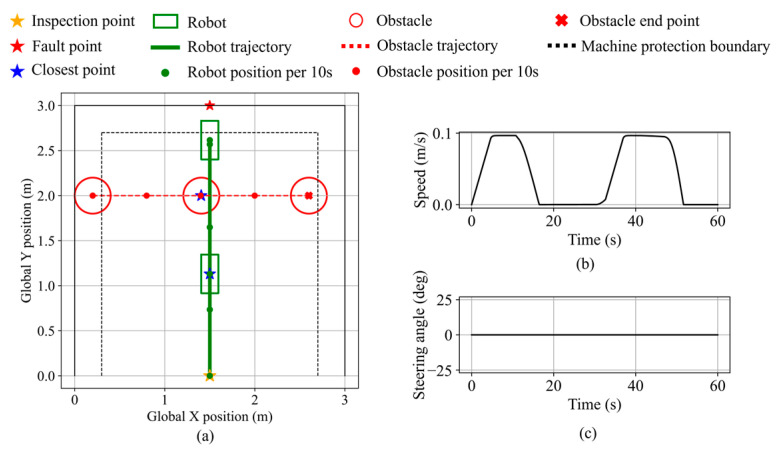
Robot states during dynamic obstacle avoidance in validation scenario 3. (**a**) Robot and dynamic obstacle trajectory. (**b**) Robot speed. (**c**) Robot steering angle.

In scenario 3, a dynamic obstacle moves laterally across the robot’s path from the front-left. The inspection and fault points are set at (1.5, 0) and (1.5, 3.0), respectively. As shown in Figure 31, the avoidance behavior is triggered, and braking is applied to ensure a smooth response, thereby improving system stability by avoiding abrupt turns. The minimum distance to the obstacle is 38.4 cm, the minimum distance to the equipment is 16.9 cm, and the final distance from the robot’s front center to the fault point is 16.9 cm.

**Figure 32:**
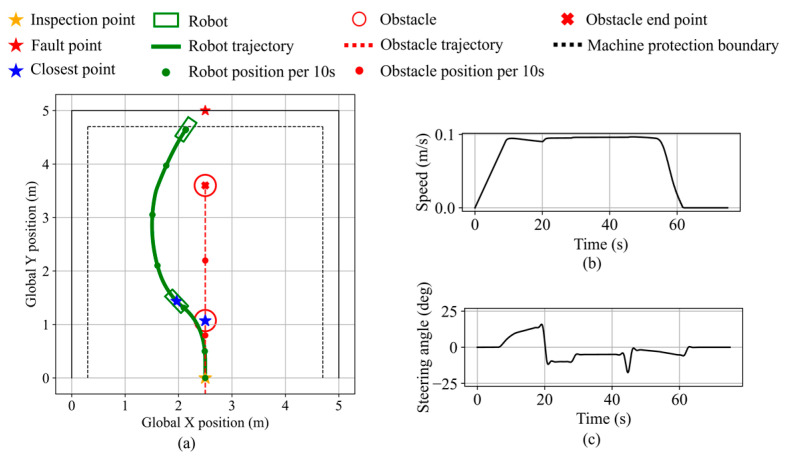
Robot states during dynamic obstacle avoidance in validation scenario 4. (**a**) Robot and dynamic obstacle trajectory. (**b**) Robot speed. (**c**) Robot steering angle.

The response to a rear-approaching dynamic obstacle is validated in scenario 4. The simulation area is a 5 m square, with the inspection and fault points set at (2.5, 0.0) and (2.5, 5.0), respectively. As shown in Figure 32, upon triggering avoidance, the robot performs a left turn, maintaining a minimum clearance of 22.1 cm. As the obstacle continues forward along the robot’s right side, the relative motion indicates a receding trend, leading the robot to resume navigation. When the obstacle becomes stationary with low blockage of the fault point, the robot maintains navigation mode and proceeds to the equipment boundary, stopping with a minimum distance of 10.2 cm from the equipment. The final distance from the robot’s front center to the fault point is 27.8 cm.

**Figure 33:**
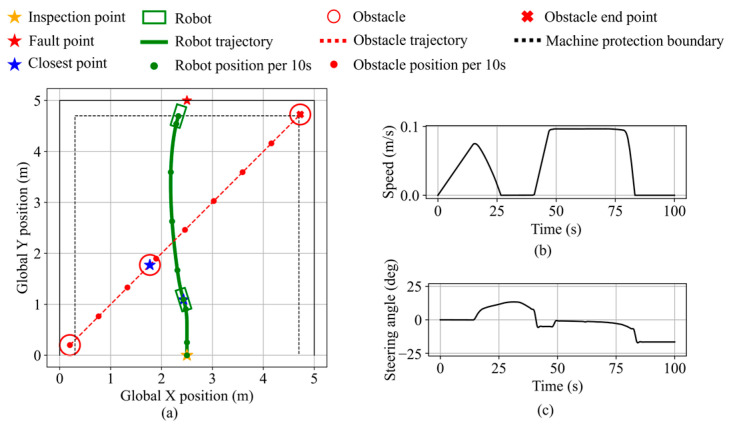
Robot states during dynamic obstacle avoidance in validation scenario 5. (**a**) Robot and dynamic obstacle trajectory. (**b**) Robot speed. (**c**) Robot steering angle.

The response to a rear-left approaching dynamic obstacle is validated in scenario 5. The factory environment is identical to scenario 4, with the obstacle and robot starting simultaneously. As shown in Figure 33, upon triggering the obstacle avoidance function, the robot executes a left turn combined with braking to avoid the obstacle, maintaining a minimum distance of 51.4 cm from the obstacle. Finally, the robot stops along the equipment protection boundary, with a minimum distance of 7.4 cm from the equipment. The final distance from the robot’s front center to the fault point is 14.6 cm.

**Figure 34:**
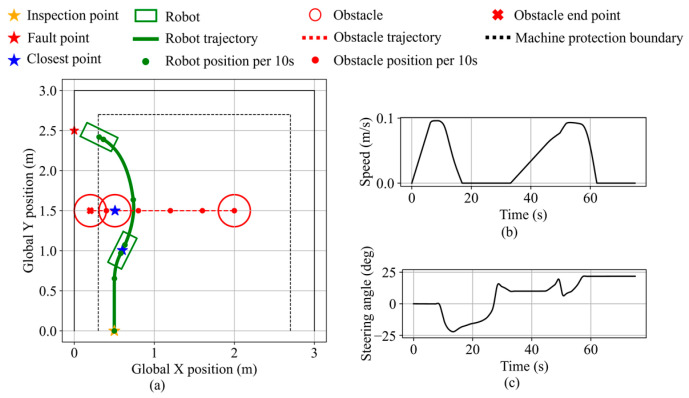
Robot states during dynamic obstacle avoidance in validation scenario 6. (**a**) Robot and dynamic obstacle trajectory. (**b**) Robot speed. (**c**) Robot steering angle.

The dynamic obstacle avoidance behavior near equipment is validated in scenario 6. The environment is a 3 m square area with the inspection and fault points located at (0.5, 0.0) and (0.0, 2.5), respectively. A dynamic obstacle moves laterally in front of the robot from the front-right. As shown in Figure 34, upon triggering the avoidance function, the robot steers away from the equipment and applies braking. The minimum distance from the obstacle is 7.9 cm. After the obstacle stops in front of the left-side equipment and its blocking level decreases, the robot resumes navigation and stops along the equipment protection boundary, maintaining a minimum distance of 7.6 cm from the equipment. The final distance from the robot’s front center to the fault point is 11.8 cm.

### 6.4. Multi-Objective Planning Validation

**Figure 35:**
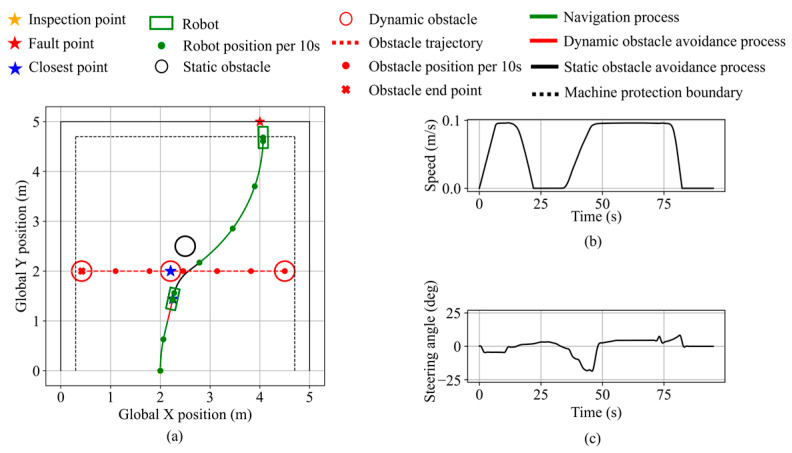
Robot states during multi-objective planning in validation scenario 1. (**a**) Robot and dynamic obstacle trajectory. (**b**) Robot speed. (**c**) Robot steering angle.

To validate the capability for multi-objective motion planning of the proposed algorithm, a scenario integrating navigation, static obstacle avoidance, and dynamic obstacle avoidance is constructed. The inspection and fault points are set at (2.0, 0.0) and (4.0, 5.0), respectively. A static obstacle is placed at (2.5, 2.5), and a dynamic obstacle moves along the line y = 2.0 from the right, starting simultaneously with the robot. As shown in Figure 35, the robot initially performs navigation toward the fault point as the dynamic obstacle remains outside the safety boundary. As the dynamic obstacle approaches, its relative speed expands the dynamic safety boundary, triggering avoidance based on its increasing urgency. The robot decelerates to avoid the dynamic obstacle, maintaining a minimum distance of 12.8 cm. Once the dynamic obstacle recedes, the static obstacle begins blocking the fault direction. Based on the updated urgency inference, the robot turns right to avoid the static obstacle, keeping a minimum distance of 14.0 cm. When the obstruction level decreases, the avoidance demand drops, and the robot resumes navigation. It stops along the equipment protection boundary, with a minimum distance of 10.2 cm to the equipment. The final distance from the robot’s front center to the fault point is 12.1 cm.

**Figure 36:**
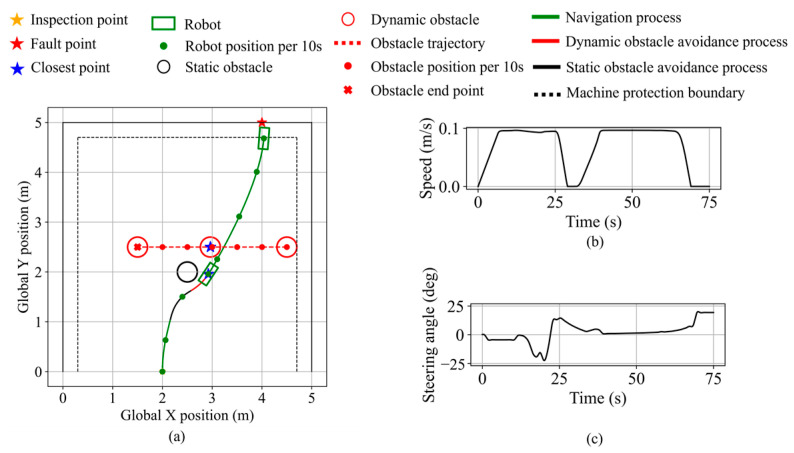
Robot states during multi-objective planning in validation scenario 2. (**a**) Robot and dynamic obstacle trajectory. (**b**) Robot speed. (**c**) Robot steering angle.

A comparative test is constructed in which a static obstacle is placed at (2.5, 2.0), closer to the robot’s initial position, while a dynamic obstacle is positioned behind it, moving from right to left along y = 2.5 and starting simultaneously with the robot. As shown in Figure 36, the robot first avoids the static obstacle. During this process, the dynamic obstacle approaches the robot, and the fuzzy system continuously evaluates the urgency inference of both obstacles. At a certain point, the dynamic obstacle is assessed as more urgent, prompting the robot to initiate avoidance of it. The minimum distances maintained from the static and dynamic obstacles are 6.5 cm and 11.1 cm, respectively. The results demonstrate that the system does not rely solely on static or dynamic distinction; rather, it evaluates both types of obstacles against urgency metrics to determine the most critical avoidance target. The robot stops along the equipment protection boundary, maintaining a minimum distance of 9.7 cm from the equipment. The final distance from the robot’s front center to the fault point is 11.8 cm.

## 7. Conclusions

To address the limited consideration of safety factors during fault point localization in diagnostic robots, a hierarchical FLC system is proposed in this study. The system emphasizes equipment protection, obstacle avoidance for both static and dynamic obstacles, and balanced coordination among multiple objectives. Based on practical application scenarios, three subfunctions were developed using zero-order T–S FLC, including navigation, static obstacle avoidance, and dynamic obstacle avoidance. In the navigation module, both equipment distance and orientation were considered to achieve coordinated navigation and protection. For obstacle avoidance, the commonalities and differences between static and dynamic obstacles were analyzed. A dynamic safety boundary was introduced, and normalized breached level was used to replace traditional distance inputs, thereby unifying the decision criteria. In the dynamic obstacle avoidance module, the influence of relative speed direction was additionally considered. All submodules were designed with equipment protection constraints. A Mamdani-type FIS was applied to infer both the necessity and urgency of avoidance, enabling coordination between navigation and avoidance functions as well as prioritization of the obstacle to be avoided. Simulation results demonstrated that the proposed system could safely guide the robot to within 30 cm of the fault point while ensuring protection of both the robot and surrounding equipment.

Future research will focus on enhancing both the adaptability and precision of the proposed system. First, parameter optimization strategies, such as evolutionary algorithms or reinforcement learning techniques, will be investigated to enable adaptive tuning of the membership functions in response to varying environmental conditions. Second, the fuzzy rule base will be systematically refined by using data-driven and clustering-based methods, such as Fuzzy C-Means Clustering and Self-Organizing Map. Moreover, efforts will be made to optimize the computational efficiency of the algorithm for embedded hardware deployment, thereby ensuring real-time responsiveness. Finally, the proposed system will be validated through implementation on an actual robotic platform, providing practical evidence of its effectiveness and reliability.

## Figures and Tables

**Table 1:** Navigation steering angle output rule base.

Δ*θ*_fault-boundary_	*θ*_fault_						
	Z	SP	SN	MP	MN	HP	HN
L	0	+L1	−L1	+L2	−L2	+L3	−L3
M	0	+L3	−L3	+L4	−L4	+L5	−L5
S	0	+L4	−L4	+L5	−L5	+L6	−L6

**Table 2:** Navigation-traction-acceleration-output rule base.

*d*_boundary_	Δ*θ*_fault-boundary_	*θ*_fault_						
		Z	SP	SN	MP	MN	HP	HN
F	L/M/S	P6	P5	P5	P4	P4	P3	P3
M	L/M/S	P5	P4	P4	P3	P3	P2	P2
N	L	P2	P2	P2	P2	P2	P2	P2
N	M/S	P4	P3	P3	P2	P2	P1	P1
VN	L	P1	P1	P1	P1	P1	P1	P1
VN	M/S	N4	N3	N3	N2	N2	N1	N1

**Table 3:** Static obstacle-avoidance steering-angle-output rule base.

*d*_boundary_	*θ*_obs_						
	Z	SP	SN	MP	MN	HP	HN
F, M	sgn(*θ*_fault_) × L3	−L2	L2	−L1	L1	0	0
N, VN	−sgn(*θ*_boundary_) × L3	−L2	L2	−L1	L1	0	0

where sgn(*θ*_fault_) and sgn(*θ*_boundary_) denote the directional signs of the fault point azimuth and the azimuth of the nearest boundary equipment, respectively.

**Table 4:** Static obstacle-avoidance traction-acceleration-output rule base.

*d*_boundary_	Δ*θ*_fault-boundary_	*θ*_sta_obs_						
		Z	SP	SN	MP	MN	HP	HN
F, M	/	P2	P3	P3	P4	P4	P5	P5
N	/	P1	P2	P2	P3	P3	P4	P4
VN	S, M	N1						
VN	L	P1	P2	P2	P3	P3	P4	P4

**Table 5:** Dynamic obstacle-avoidance steering-angle-output rule base.

*d*_boundary_	*θ*_dyn_obs_	*θ*_obs_v_							
		F	B	FL	L	BL	FR	R	BR
F, M	F, B	sgn(*θ*_fault_) × L3	sgn(*θ*_fault_) × L3	−L3	0	L3	L3	0	−L3
	FL	0	0	0	0	0	L2	0	−L2
	BL	0	0	0	0	0	L2	0	0
	L	0	0	0	0	0	L1	0	0
	FR	0	0	−L2	0	L2	0	0	0
	BR	0	0	−L2	0	0	0	0	0
	R	0	0	−L1	0	0	0	0	0

where sgn(*θ*_fault_) denotes the directional signs of the fault point azimuth.

**Table 6:** Dynamic obstacle-avoidance steering-angle-output rule base during equipment protection.

*d*_boundary_	*θ*_obs_v_							
	F	B	FL	BL	FR	BR	L	R
N, VN	−sgn(*θ*_boundary_) × L1		−sgn(*θ*_boundary_) × L2				−sgn(*θ*_boundary_) × L3	

where sgn(*θ*_boundary_) denotes the azimuth of the nearest boundary equipment.

**Table 7:** Dynamic obstacle-avoidance traction-acceleration-output rule base.

*d*_boundary_	*θ*_dyn_obs_	*θ*_obs_v_							
		F	B	FL	L	BL	FR	R	BR
F, M	F, B	sgn(*θ*_fault_) × L3	sgn(*θ*_fault_) × L3	−L3	0	L3	L3	0	−L3
	FL	0	0	0	0	0	L2	0	−L2
	BL	0	0	0	0	0	L2	0	0
	L	0	0	0	0	0	L1	0	0
	FR	0	0	−L2	0	L2	0	0	0
	BR	0	0	−L2	0	0	0	0	0
	R	0	0	−L1	0	0	0	0	0

**Table 8:** Obstacle-avoidance necessity-output rule base.

Breached Level	Trend Angle	Block Angle	Necessity for Obstacle Avoidance
S	/	/	Low
PC	Approach	Block	High
	Approach	NoBlock	Med
	Recede	Block	Med
	Recede	NoBlock	Low
E	Approach	Block	VeryHigh
	Approach	NoBlock	High
	Recede	Block	Med
	Recede	NoBlock	Low

**Table 9:** Obstacle-urgency-output rule base.

Breached Level	Trend Angle	Obstacle Urgency
S	/	Low
PC	Approach	Med
	Recede	Low
E	Approach	High
	Recede	Med

**Table 10:** Parameters of RoboCar 1/10.

Parameter	Value	Unit
Mass	2200	g
Length	429	mm
Width	190	mm
Wheelbase	250	mm
Minimum radius of rotation	500	mm
Maximum speed	2.78	m/s

**Table 11:** Simulation parameters of RoboCar 1/10.

Scenarios	The Minimum Distance to the Equipment (cm)	Distance From Front Center to Fault Point (cm)
a	7.7	12.5
b	10.9	23.2
c	7.6	25.6
d	7.4	27.5
e	6.8	11.1
f	6.4	9.2

## Data Availability

The data are not publicly available due to all the simulation data being generated during the study on a local computer.

## Abbreviations

The following abbreviations are used in this manuscript:

FLC	Fuzzy Logic Control
FIS	Fuzzy Inference System
T–S	Takagi–Sugeno
